# Poly(ionic liquid)‐Armored MXene Membrane: Interlayer Engineering for Facilitated Water Transport

**DOI:** 10.1002/anie.202202515

**Published:** 2022-05-03

**Authors:** Ming Yi, Mi Wang, Yan Wang, Yanlei Wang, Jian Chang, Atefeh Khorsand Kheirabad, Hongyan He, Jiayin Yuan, Miao Zhang

**Affiliations:** ^1^ Key Laboratory of Material Chemistry for Energy Conversion and Storage Huazhong University of Science and Technology Wuhan 430074 P. R. China; ^2^ Hubei Key Laboratory of Material Chemistry and Service Failure School of Chemistry and Chemical Engineering Huazhong University of Science and Technology Wuhan 430074 P. R. China; ^3^ Department of Materials and Environmental Chemistry Stockholm University Stockholm 10691 Sweden; ^4^ Beijing Key Laboratory of Ionic Liquids Clean Process State Key Laboratory of Multiphase Complex Systems Institute of Process Engineering Chinese Academy of Sciences Beijing 100190 P. R. China

**Keywords:** Anion Exchange, Assembly, MXene, Nanochannel Hydrophilicity, Poly(Ionic Liquid)

## Abstract

Two‐dimensional (2D) MXene‐based lamellar membranes bearing interlayers of tunable hydrophilicity are promising for high‐performance water purification. The current challenge lies in how to engineer the pore wall's surface properties in the subnano‐confinement environment while ensuring its high selectivity. Herein, poly(ionic liquid)s, equipped with readily exchangeable counter anions, succeeded as a hydrophilicity modifier in addressing this issue. Lamellar membranes bearing nanochannels of tailorable hydrophilicity are constructed via assembly of poly(ionic liquid)‐armored MXene nanosheets. By shifting the interlayer galleries from being hydrophilic to more hydrophobic via simple anion exchange, the MXene membrane performs drastically better for both the permeance (by two‐fold improvement) and rejection (≈99 %). This facile method opens up a new avenue for building 2D material‐based membranes of enhancing molecular transport and sieving effect.

## Graphical abstract

The surface hydrophilicity of nanochannels within 2D MXene‐based membranes was readily engineered by poly(ionic liquid)s (PILs) as tailor‐made modifier to assemble MXene laminates via electrostatic and surpamolecular interactions. By judicious choice of exchangeable counter anion of the PILs, the interlayer galleries were precisely altered in a wide hydrophilic‐to‐hydrophobic range to amplify the nanofiltration performance.

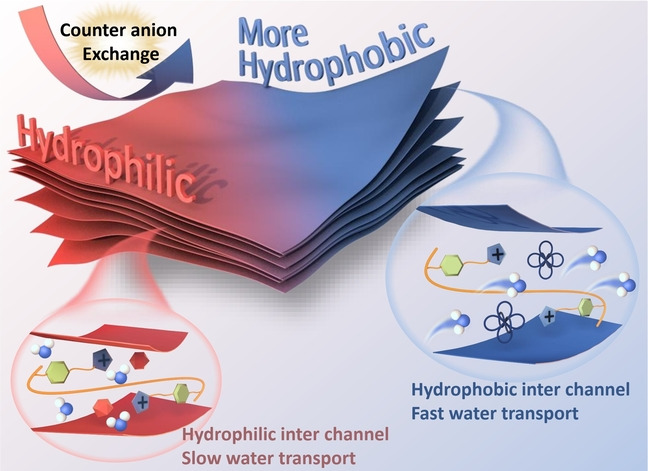

## Introduction

The discovery of two‐dimensional (2D) transition metal carbides as well as nitrides (MXenes) in 2011 has expanded the structure pool and property window of 2D materials available for a wider range of applications.^[1]^ Recently, particular interest has been raised in utilizing MXene membranes in environmental remediation and water treatment fields due to their favorable integration of characteristic properties such as being hydrophilic, remarkable mechanical strength, wet‐processability, electron conductivity and high aspect ratio.[^1b^, ^2^] Similar to other 2D materials, such as graphene and its derivatives, and transition‐metal chalcogenides, MXene‐based membranes have subnanometer interlayer channels, which offer slit‐shaped pores for water transport.^[3]^ Their sieving mechanism of target molecules is governed by size exclusion through the stacking layers and the guest–host electrostatic interaction.[^1a^, ^3b^, ^4^] In addition, the separation performance of MXene‐based membranes is sensitive to the hydrophilicity of the membrane pores when the interlayer spacing falls in a reasonably small size range. As the surface characters of membrane pores starts to determine the interaction between the passing water molecules and the pore walls, it directly dictates the water transport behavior in the confined environment.^[5]^

Generally speaking, a more hydrophilic surface of MXene endows the membrane with a strong affinity to water molecules, assisting the membrane to uptake water molecules and to increase the membrane wettability.^[6]^ Counterintuitively, both experimental observations and simulations point out that hydrophilic transport galleries go actually against water penetration since strong interaction between water molecules and pore walls increases friction and consequently reduces the flow velocity.^[7]^ In this context, membranes equipped with a hydrophilic outer surface but hydrophobic transport nanochannels are regarded optimal for speedy water purification.^[8]^ However, due to intractable construction in the subnano‐confined environment, there lacks studies so far on the effective and facile turning of pore wall's hydrophilicity of MXene‐based membranes, as MXene has been dominantly used as highly hydrophilic 2D building units. Layer‐by‐layer deposition appears as a powerful tool to construct MXene/polyelectrolyte ultrathin membranes with tunable composition and interlayer properties. However, the time‐consuming procedure mismatches the high‐throughput production required by water treatment applications. It remains a huge challenge for materials innovation and methodology revolution towards interlayer‐controllable MXene‐based membrane.^[9]^

Poly(ionic liquid)s (PILs), also termed polymerized ionic liquids, refer to a subclass of ionic polymers that feature an ionic liquid (IL) species in the repeating unit.^[10]^ The most studied PILs so far consist of a cationic polymeric backbone with its mobile counter anions.^[11]^ Counter anions play an enabling role in governing the hydrophilicity and solubility of PILs. Commonly PILs with the same cationic backbone structure are soluble in different solvents, depending on the counter anion species.^[12]^ Equally important, the anion species can be readily altered by an anion exchange reaction while the cationic backbone remains intact. These features grant PILs as multifaceted materials with a large hydrophilicity/hydrophobicity window, in our view, toward nanogallery construction of MXene‐based membranes.

**Figure 1:**
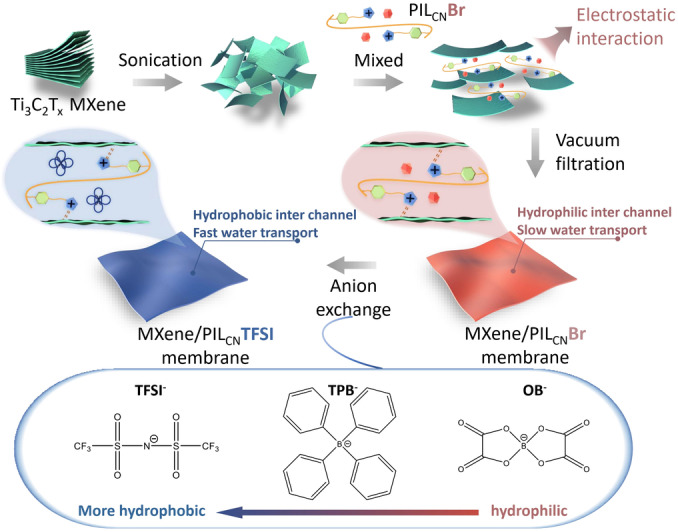
Schematic of the fabrication process of MXene/PIL membranes.

Herein, to bestow hydrophilically/hydrophobically variable nanochannels into MXene membranes, PILs were employed as tailor‐made surface modifier to assemble MXene laminates via electrostatic interactions. The anion exchange reaction was then carried out in situ to define the surface properties of pores. (Figure 1) A judicious choice of counter anions can not only endow the membrane with more hydrophobic nanochannels for better water transport, but also maintain the desirable parameters of the nanogalleries for precise sieving. This study paves the way for future development of MXene membranes as well as other ionic materials with engineerable nanochannels for target molecular transport and sieving.

## Results and Discussion

**Figure 2:**
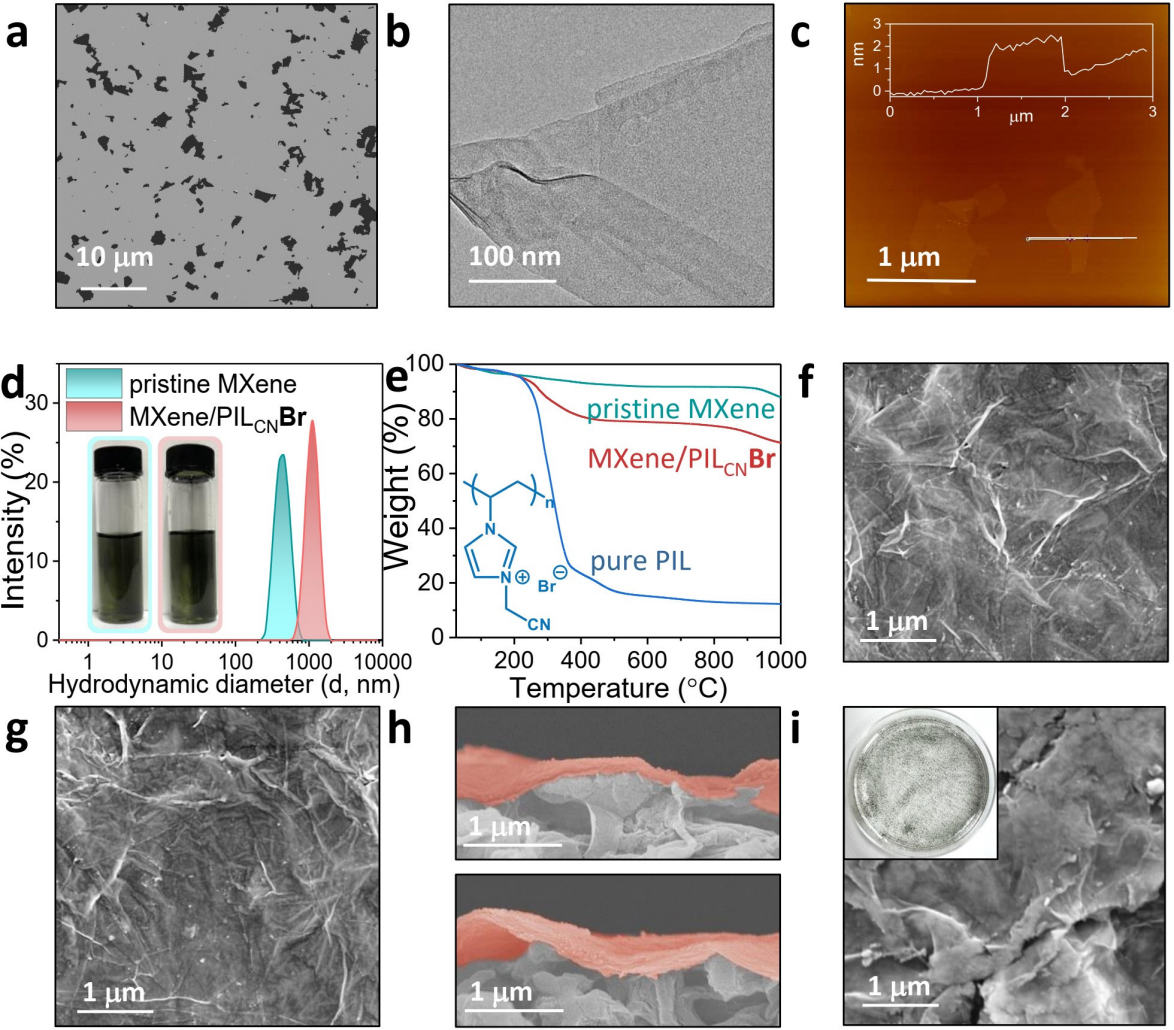
a)–c) SEM, TEM and AFM images, respectively, of pristine MXene nanosheets. d) DLS plots of the pristine MXene and the MXene/PIL_CN_ **Br** nanosheets in suspensions (insets: photographs of the corresponding dispersions in vials). e) TGA plots of pure PIL_CN_ **Br**, pristine MXene, and MXene/PIL_CN_ **Br** powers under N_2_. f), g) Surface SEM images of MXene/PIL_CN_ **Br** and MXene/PIL_CN_ **TFSI** membranes, respectively. h) Cross‐sectional SEM images of MXene/PIL_CN_ **Br** (upper) and MXene/PIL_CN_ **TFSI** (below) membranes (MXene loading amount: 160 mg m^−2^). i) Surface SEM image of MXene@PIL_CN_ **TFSI** membrane (insets: photograph of the corresponding suspension in a top view).

The MXene nanosheets were synthesized via a well‐established protocol, as described in our previous work.^[13]^ The aluminum atom layer of bulk Ti_3_AlC_2_ was etched away in a LiF/HCl solution with Li^+^ ions being intercalated simultaneously. The as‐synthesized MXene nanosheets were first characterized jointly by scanning electron microscopy (SEM, Figure 2a), transmission electron microscopy (TEM, Figure 2b), and atomic force microscopy (AFM, Figure 2c). The corresponding results consistently indicated that the nanosheets featured a lateral size of 0.5–4 μm and a height of 1.5–2 nm, with a smooth surface. Considering the theoretical thickness of ≈1.0 nm for monolayer MXene and its surface bound water layer,^[14]^ the as‐synthesized MXene was regarded as monolayer nanosheets.^[15]^ The structural integrity of MXene nanosheets free of impurity and oxidation was identified by high‐resolution transmission electron microscope (HRTEM) image (Figure S1). Furthermore, clear lattice fringes of the Ti atom layer shown in the selected area electron diffraction (SAED) patterns indicated the well‐reserved hexagonal symmetry inherited from the bulk Ti_3_AlC_2_ powder.^[16]^ The element mapping results in the energy‐dispersive X‐ray spectroscopy (EDX) images of dried MXenes showed a homogeneous distribution of Ti, C, O, and F elements in the MXene nanosheets (Figure S2). The X‐ray diffraction (XRD) patterns (Figure S3) acquired from the dried sample consistently support the successful transformation of the MAX phase into MXene nanosheets, as the peak located at 39° assigned to the (104) plane disappeared and the diffraction peak for the (002) plane downshifted after etching.^[17]^ All the above results confirmed the successful etching of MAX, and the delamination of MXene nanosheets.

PILs of different chemical structures were designed and employed to construct the hydrophilicity‐tunable galleries of MXene nanosheets. Specifically speaking, a hydrophilic cationic PIL poly(1‐vinyl‐3‐cyanomethylimidazolium bromide) (termed PIL_CN_ **Br**, where CN refers to the distal group in the substituent of the polyimidazolium backbone, **Br** denotes the counter anion, chemical structure seen in Figure S4) was used as building units in the ionic deposition. Briefly, a PIL_CN_ **Br** aqueous solution was first added into a MXene suspension and the mixture was stirred for 12 h to complete modification and assembly of MXene nanosheets. The PIL‐tethered MXene nanosheets was denoted as MXene/PIL_CN_ **Br** hereafter. The AFM image of MXene/PIL_CN_ **Br** nanosheets showed an increased thickness of 2.5–3 nm as compared to the pristine MXene nanosheets (1.5–2 nm) (Figure S5). As mentioned above, the theoretical thickness is ≈1.0 nm for a monolayer MXene with a bound water layer on its surface. Thus the PIL chains were believed to electrostatically attach to both flat surfaces of the MXene sheets. Besides, the pristine MXene held a mean particle size of 560±14 nm, whilst the MXene/PIL_CN_ **Br** delivered a nearly doubled mean particle size of 1247±38 nm (Figure 2d and S6). It indicated the successful assembly of MXene nanosheets with positively charged PIL_CN_ **Br**. Note that no optical sedimentation occurred in the mixture suspension (digital photograph in Figure 2d), the well‐dispersed state is key to form the crack‐free top layer during the subsequent membrane fabrication. Accordingly, the MXene/PIL_CN_ **Br** suspension after washing treatment was measured to have a positive Zeta potential value of +23.9±1.1 mV at pH 7 (Figure S7). In comparison, a polymer‐free MXene suspension gives a negative value of −39.7±0.6 mV, indicative of successful surface charge reversion during this modification. In addition, the laminar structure and the thickness of the MXene membrane were maintained consistently after assembly by PIL_CN_ **Br** (Figure S8). The content of PIL_CN_ **Br** in MXene/PIL_CN_ **Br** nanosheets was calculated to be 16.9 wt % according to the thermal gravimetric analysis (TGA, Figure 2e). Next, the pristine MXene and MXene/PIL_CN_ **Br** membranes were prepared by filtering the corresponding suspensions through a polydopamine‐coated porous nylon‐66 substrate with an average pore size of 0.22 μm (Figure 1). To note, the cationic PIL_CN_ chains are associated with the anionic parts of MXene nanosheets through electrostatic interactions and supramolecular interactions (e.g., van der Waals interactions between PIL_CN_ and the surface groups of MXene). Such interplays favor the trapping of PIL_CN_ chains into stacked MXene nanosheets, endowing the membrane with extra MXene‐PIL channels for molecular transport. By depositing counter anions in these MXene‐PIL channels, the surface properties of the MXene interlayer are expected to be precisely manipulated, as discussed in detail later.

In a final step, Br^−^, the counter anion of PIL_CN_ **Br** was in situ exchanged by flowing a solution containing a different counter anion throughout the MXene/PIL_CN_ **Br** membrane. Here three counter anions, including hydrophilic bis(oxalate)borate (OB^−^) (chemical structures and anion exchange mechanism presented in Figure S4) and two more hydrophobic ones, i.e., the bis(trifluoromethylsulfonyl)imide (TFSI^−^) and tetraphenylborate (TPB^−^), were applied for a systematical investigation. The corresponding membranes were referred to as MXene/PIL_CN_ **TFSI**, MXene/PIL_CN_ **OB**, and MXene/PIL_CN_ **TPB**, respectively. As control experiment, TFSI^−^ was added directly into a MXene/PIL_CN_ **Br** mixture dispersion before the membrane preparation, i.e., an ex situ approach, and the mixture dispersion with certain emerging coagulation was sonicated for homogenization and then filtration‐deposited onto the same porous substrate. The product was termed MXene@PIL_CN_ **TFSI**.

The surface morphology of the reference membrane was characterized and analyzed. For both pristine MXene and the MXene/PIL_CN_ **Br** membranes, no visible cracks or defects were observed on the wrinkled membrane surfaces, suggesting a uniform deposition of MXene nanosheets onto the support (Figure 2f and Figure S9). The uniform distribution of N element across the membranes further indicated the homogeneous attachment of PIL_CN_ **Br** chains onto MXene nanosheets (Figure S10). The cross‐sectional image showed that the MXene/PIL_CN_ **Br** membrane held a well‐organized lamellar morphology with an average thickness of 225±22 nm (Figure 2h). These results indicated a uniform, nanoscale and integrated layer of MXene/PIL_CN_ **Br** on the support. After the anion exchange, the surface integrity and the well‐packed 2D lamellar morphology of the membranes were maintained well, as observed in the SEM image of MXene/PIL_CN_ **TFSI** (Figure 2g, h). The macroscopic membrane images also showed an integrated MXene layer with the anion exchange of TFSI^−^ without visible defects (Figure S11). In addition, the evenly distributed S element (originated from the TFSI^−^ counter anion) in the corresponding EDS element mapping images of both surface and cross‐sectional showcased the homogeneous anion distribution across the entire membranes (Figures S10 and S12). Likewise, the surface EDS mapping for MXene/PIL_CN_ **OB** and MXene/PIL_CN_ **TPB** presented a similar distribution pattern of B element as well. In comparison, as conducted in the control experiment, when the MXene and PIL_CN_ **Br** mixture dispersion was directly exposed to TFSI^−^ before the membrane preparation, sedimentation occurred and insoluble aggregates appeared in the MXene@PIL_CN_ **TFSI** suspension (Figure S13). The same phenomenon was observed in both MXene@PIL_CN_ **OB** and MXene@PIL_CN_ **TPB** samples. For MXene@PIL_CN_ **TFSI** and MXene@PIL_CN_ **TPB**, the precipitate of hydrophobic PIL_CN_ **TFSI** and PIL_CN_ **TPB** from the aqueous solutions led to the sedimentation of MXene/PIL nanosheets bearing the corresponding hydrophobic anions.^[18]^ In the case of MXene@PIL_CN_ **OB**, the OB^−^ anion, though much less hydrophobic than the former two anions, is still more hydrophobic enough than Br^−^ to precipitate PIL_CN_ **OB** out of water. As a result, MXene attached by the PIL_CN_ **Br** backbone also sedimented when mixing with OB^−^. As a result, deep wrinkles and visible cracks along edges of MXene nanosheets were observed in the SEM image of MXene@PIL_CN_ **TFSI** (Figure 2i), indicating its non‐dense, fragmentized surface with large holes as defects. Such defects are detrimental to separation of objects smaller than the holes. Our structural analyses here manifested the superiority of the anion exchange process when constructing MXene‐based membrane with hydrophobic PILs in an aqueous phase. It satisfied the homogeneous dispersion of MXene in a monolayer state and ensured the integrity of the MXene membrane, while circumventing the defects formation due to the sedimentation of MXene laminates.

**Figure 3:**
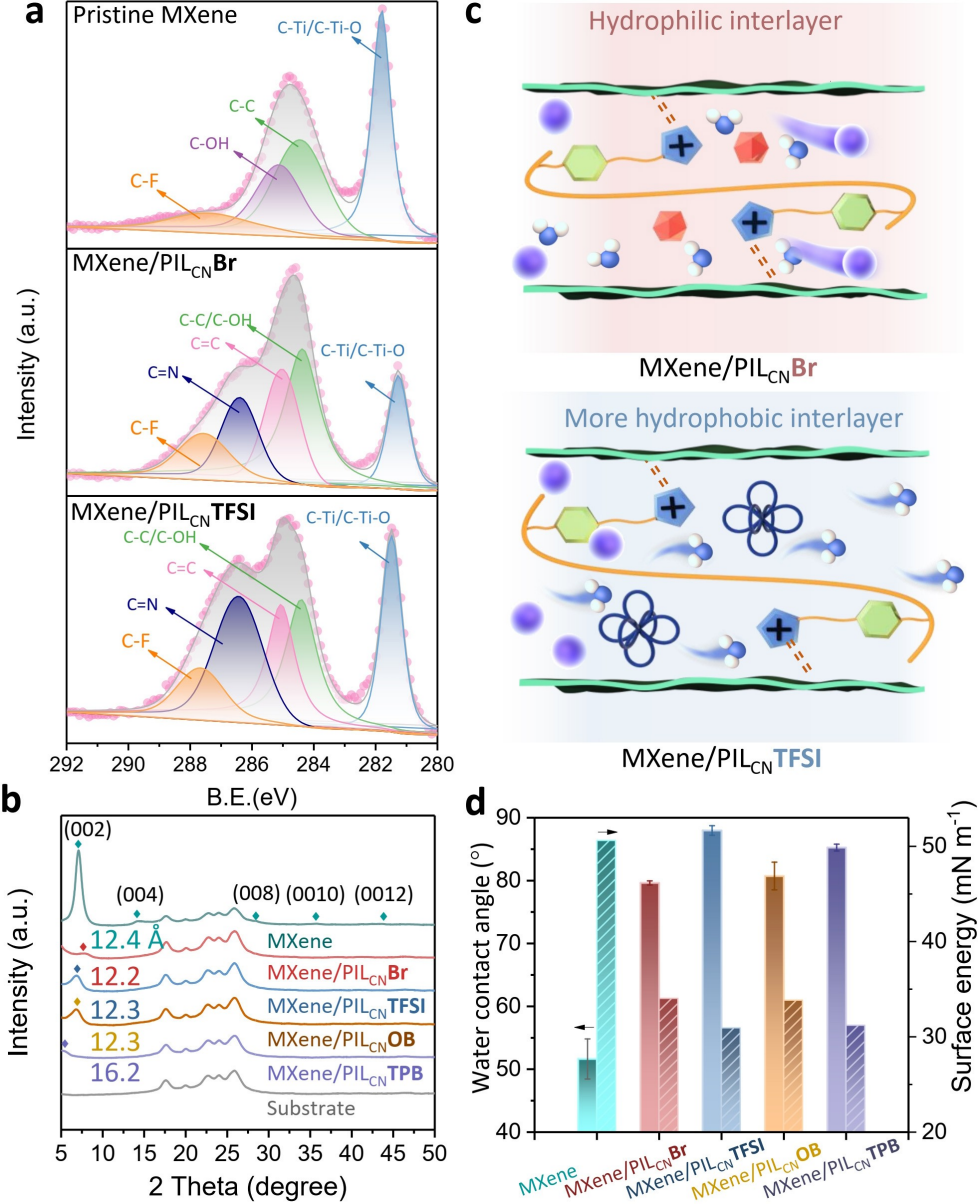
a) Peak deconvolution of the narrow‐scan spectra of C 1s for pristine MXene, MXene/PIL_CN_ **Br**, and MXene/PIL_CN_ **TFSI** membranes. b) XRD results of pristine MXene, MXene/PIL_CN_ **Br** and various anion‐exchanged MXene membranes. c) Schematic illustration of anion exchange in MXene/PIL laminates. d) Water contact angle and surface energies of pristine MXene, MXene/PIL_CN_ **Br** and various anion‐exchanged MXene membranes.

To investigate the chemistry behind the assembly of MXene by PILs, as well as the anion exchange, the feature elements were traced by X‐ray photoelectron spectroscopy (XPS) analysis and EDS mapping. Unsurprisingly, the existence of N element was detected from PIL_CN_‐assembled MXene membranes (Table S1 and S2), as it is the defining characteristic of the polymer. In addition, the peaks at 284.1 and 286.5 eV, assigned to C=C and C=N species, respectively, confirmed the presence of the imidazolium moiety of PIL_CN_ (Figure 3a and Figure S14).^[19]^ Following the anion exchange, the amount of F in the MXene/PIL_CN_ **TFSI** membrane increased from 5.89 % (atomic percentage of F in pristine MXene) to 8.96 % (atomic percentage of F coming from both MXene and the attached PIL_CN_ **TFSI**), which evidenced the successful occurrence of anion exchange reaction from Br^−^ to F‐rich TFSI^−^ anion (Table S1). In addition, the MXene in the MXene/PIL_CN_ **TFSI** composite showed slight oxidation, as a weak peak located at 459.04 eV associated with TiO_2_ appeared (Figure S15).^[20]^

To figure out the effect of anion exchange on the lamellar microstructure of the MXene layer, the interlayer spacing (*d*‐spacing) of MXene membranes before and after anion exchange was characterized by XRD (Figure 3b). For the pristine MXene membrane, the (002) peak located at 7.1° corresponds to a *d*‐spacing of 1.24 nm.^[4]^ After the assembly of MXene by PIL_CN_ **Br**, the MXene/PIL_CN_ **Br** membranes show a narrowed nanochannels size of 1.21 nm, which is consistent with the recent literature.^[2b]^ By replacing Br^−^ with a larger anion in MXene/PIL nanochannels, expectedly, the interlayer distance in MXene laminates was observed to expand only slightly to 1.23 nm for both MXene/PIL_CN_ **TFSI** and MXene/PIL_CN_ **OB**, which is quite close to the *d*‐spacing of the pristine MXene membrane (1.24 nm). These similar interlayer spacings guaranteed the hydrophilicity of the nanogallery as the sole variable of counter anion exchange. Notably, MXene/PIL_CN_ **TPB** with observably larger anion possessed a significantly higher *d*‐spacing value of 1.62 nm, and such wide galleries were unfavorable for effective retention of target solutes smaller than the *d*‐spacing.

Generally speaking, the intrinsic hydrophilicity of nanochannels in MXene/PIL_CN_ **Br** membranes could be effectively altered by the substitution of Br^−^ with the more hydrophobic TFSI^−^ anion, as illustrated in Figure 3c. The water contact angles were measured and the corresponding surface energy was then calculated, which reflects the intrinsic hydrophilicity feature of materials. As shown in Figure 3d, the pristine MXene membrane possessed a super hydrophilic surface with a water contact angle of as low as 51.6° and a relatively high surface energy of 50.6 mN m^−1^, which could be ascribed to the hydroxyl‐rich surface of MXene nanosheets. In comparison, the membrane surface became less hydrophilic when the PIL chains were immobilized to the MXene surface. Then the surface energy further decreased to ≈31 mN m^−1^ after the anion exchange of Br^−^ into TFSI^−^ or TPB^−^ with water contact angles in the range of 85.3–88.0°. These results confirmed the concept of manipulating membrane surface properties via anion exchange. To note, the surface became only less hydrophilic (<90°) instead of being hydrophobic (>90°), ensuring still reasonably good wetting of water molecules to the MXene/PIL_CN_ **TFSI** surface, which was the premise for fast water permeation through membranes.[^5^, ^8^]

**Figure 4:**
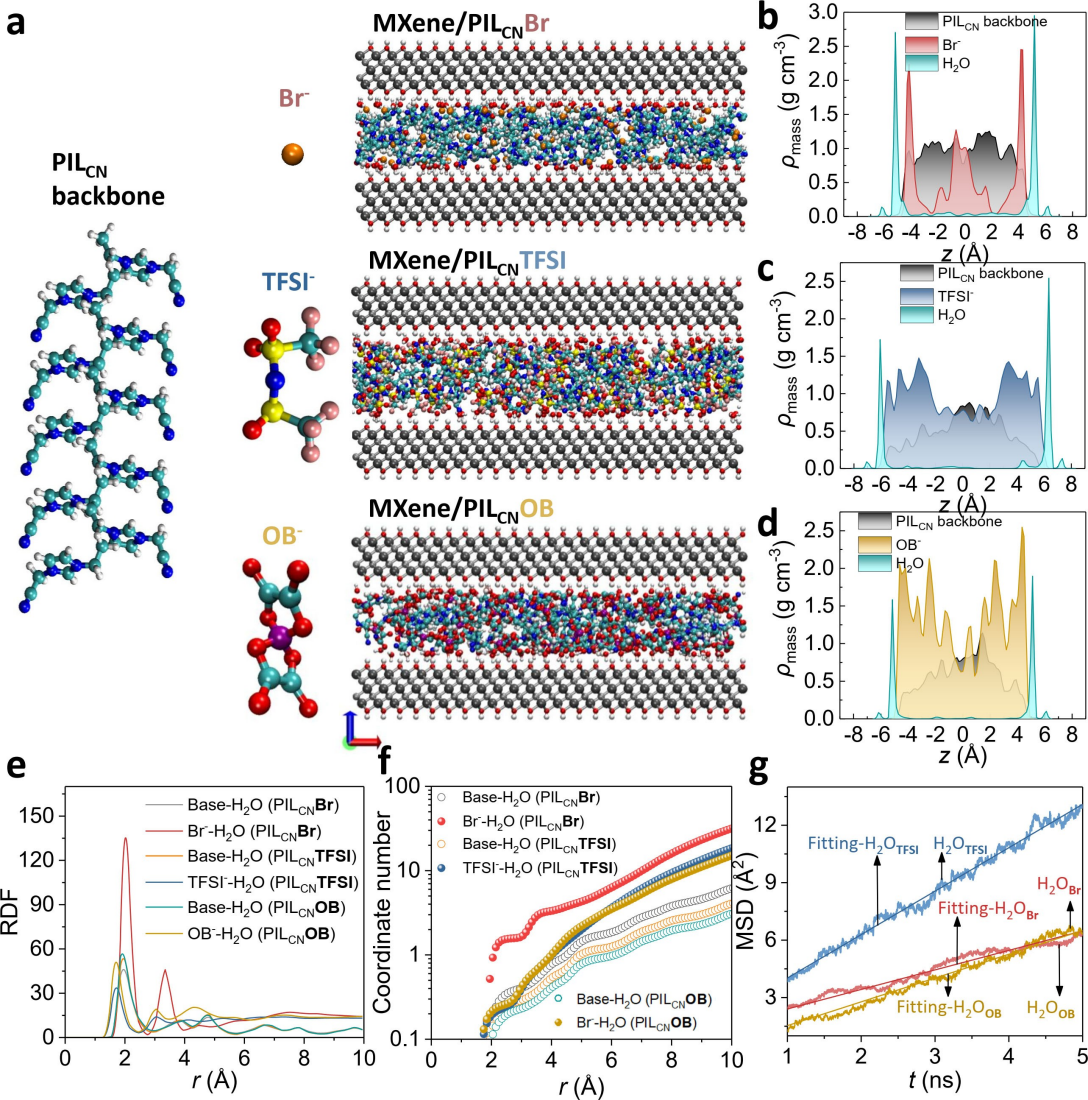
a) Atomic structures of the water, PIL_CN_ backbone and anions in the MD simulation model. Atomic mass density distribution of the water, PIL_CN_ backbone and anions in the nanochannel for b) MXene/PIL_CN_ **Br**, c) MXene/PIL_CN_ **TFSI** and d) MXene/PIL_CN_ **OB**. e) The radial distribution function (RDF) and f) coordinate number distribution between water and anion/MXene. g) Mean‐square‐distance (MSD) and self‐diffusion coefficient of water molecules in the nanochannel for different PILs.

In addition to surface hydrophilicity, the nanochannels’ affinity towards water molecules determines the water diffusion and transport through the MXene laminates. To pinpoint the intrinsic role of PILs on the diffusive behavior of water in the nanochannels in PIL‐assembled MXene, molecular dynamics (MD) simulations were performed. As shown in Figure 4a–d, the nanochannels of MXene/PIL_CN_ **Br**, MXene/PIL_CN_ **TFSI**, and MXene/PIL_CN_ **OB** were constructed by placing MXene nanosheets and PILs according to the structure model of a sandwich. Figure 4b, c and d display the atomic mass density for polycation, anion, and water molecules. Obviously, the PILs occupied most of the spaces in the nanochannel. Interestingly, the water molecules preferred the distribution in the interfacial region between MXene and PIL chains rather than the gap within the PILs. Meanwhile, the radial distribution function (RDF) and coordination number between water and PILs were also calculated, respectively (Figure 4e and f). The intensity of the first peak in RDF and coordination number for water‐Br^−^, water‐OB^−^ and water‐TFSI^−^ was 154.2, 57.0, and 36.98, respectively. The coordination number for water around various anions follow the same order as the intensity of RDF. The results indicated the stronger interaction between water and MXene/PIL_CN_ **Br** than MXene/PIL_CN_ **OB** or MXene/PIL_CN_ **TFSI**, which agreed well with the contact angle measurements and the surface energy results shown in Figure 3d. Such difference in the water affinity between Br^−^ and TFSI^−^ affects the water transport behavior in the MXene nanochannels. To probe the diffusive ability of water molecules, the mean‐square‐distance (MSD) of water molecules confined in nanochannels was further obtained (Figure 4g), where the self‐diffusion coefficient for water in MXene/PIL_CN_ **Br**, MXene/PIL_CN_ **OB** and MXene/PIL_CN_ **TFSI** were 2.55, 3.11 and 5.67×10^−12^ m^2^ s^−1^, respectively. This indicated that water molecules diffused twice faster in the nanochannels of MXene/PIL_CN_ **TFSI** than that of MXene/PIL_CN_ **Br**, which is beneficial for efficient water purification and molecular sieving.

To pinpoint the effect of the assembly of MXene by PILs and the subsequent anion exchange step on the practical separation performance, the permeances and rejections of resultant membranes were evaluated through the nanofiltration process using a lab‐scale cross‐flow nanofiltration setup under an operational pressure of 1 bar. The loading of the MXene/PIL_CN_ **Br** nanosheets on the support layer was firstly optimized. Similar to other 2D material‐based membranes,^[21]^ a larger amount of loading of MXene nanosheets gave a higher rejection and a lower permeance to the MXene/PIL_CN_ **Br** membranes (Figure 4a). Given the thicker layer caused by a larger amount of MXene loading (Figure S16), it brought more significant resistance of mass transfer but less defects. Specifically, when the loading of MXene exceeded 80 mg m^−2^, the MXene/PIL_CN_ **Br** membrane reached a rejection higher than 96.7 % for the targeted solute Congo Red (CR). Considering the trade‐off between water permeance and rejection, the loading contents of 80 and 160 mg m^−2^ were chosen as optimal values for the separation tests discussed below.

As discussed above, the effective anion exchange process altered the hydrophilicity of the surface and nanochannels of the assembled MXene/PIL membranes. Primarily, the stability of counter anions in the MXene interlayer during the filtration tests was investigated. In detail, the MXene/PIL_CN_ **TFSI** membrane was first filtrated with NaCl aqueous solution (100 ppm) for 30 min (at 1 bar) and then was digested. By inductively coupled plasma optical emission spectrometry (ICP‐OES) analysis, the S content was found well‐maintained before and after the filtration tests (Figure S17). This result suggested the satisfactory stability of counter anions in the MXene layer during the filtration tests, a prerequisite for hydrophilicity tailoring of nanogallaries for MXene/PIL membranes.

**Figure 5:**
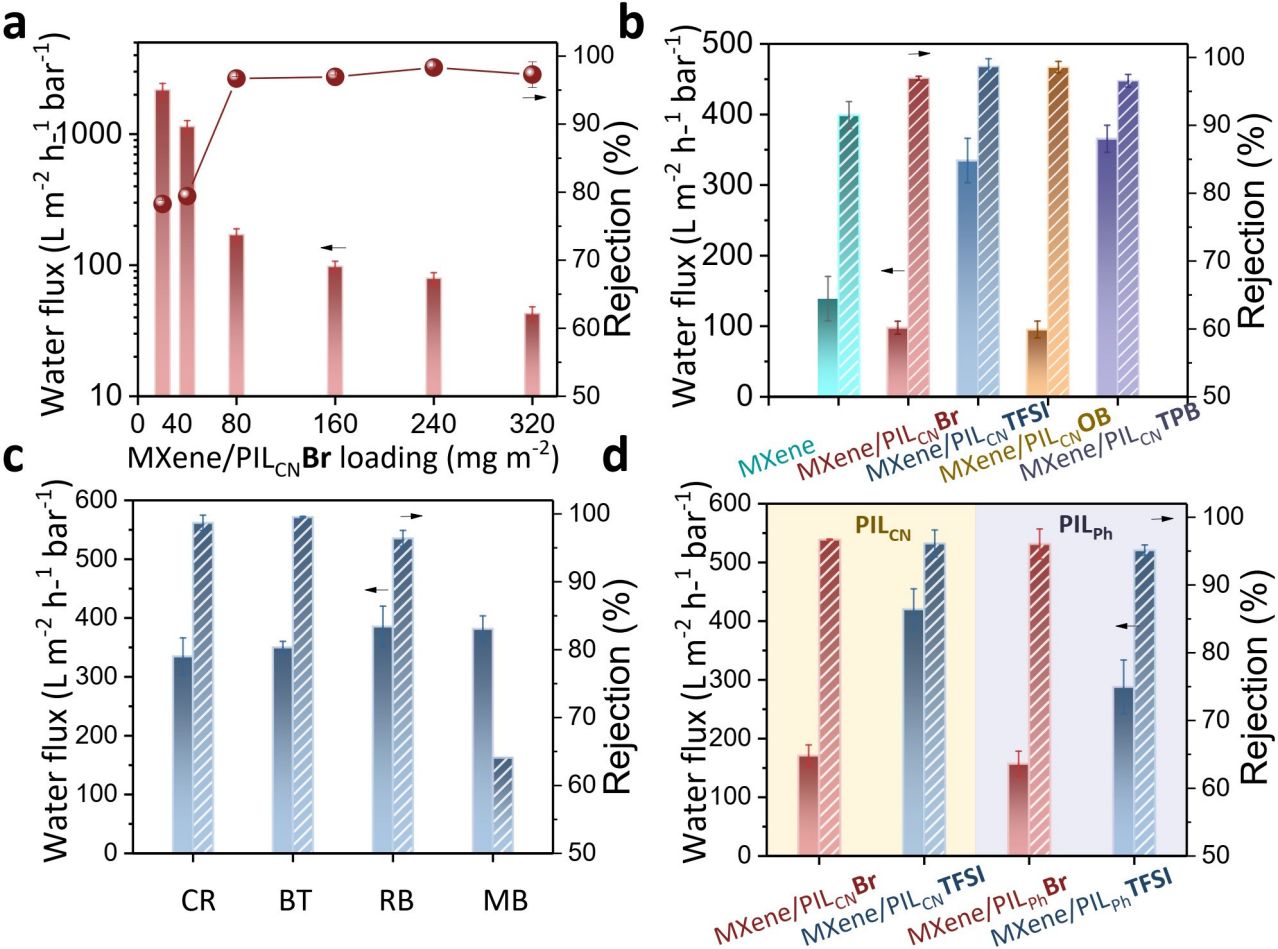
a) The effect of MXene/PIL_CN_ **Br** loading on the separation performance of the MXene/PIL_CN_ **Br** membrane. Separation performance of b) MXene, MXene/PIL_CN_ **Br** and various anion‐exchanged MXene membranes to CR (MXene loading: 160 mg m^−2^); c) MXene/PIL_CN_ **TFSI** membrane for solutions containing various dye molecules (MXene loading: 80 mg m^−2^). CR: Congo Red, molecular weight (MW) of 696.66 g mol^−1^; BT: Eriochrome Black T, MW of 461.38 g mol^−1^; MB: Methylene Blue, MW of 319.85 g mol^−1^; RB: Rose Bengal, MW of 1017.64 g mol^−1^; d) various PILs‐assembled MXene membranes before and after anion exchange (MXene loading: 160 mg m^−2^).

Effects of anion exchange process on the transport and passage of water molecules further impacted the membrane's separation performance. As clearly seen from Figure 5b, the pristine MXene membrane at a loading content of 160 mg m^−2^ held a permeance of 139.0 L m^−2^ h^−1^ bar^−1^ and an unsatisfactory CR rejection of 91.5 %. In comparison, at the same loading content, the permeance decreased to 97.8 L m^−2^ h^−1^ bar^−1^ with an improved rejection of 96.9 % for MXene/PIL_CN_ **Br**, due to the decreased affinities of the pore wall surface to water (Figure 3c) and narrowed interlayer galleries of the MXene layer (Figure 3b). After anion exchange to replace Br^−^, it was interestingly found that the MXene/PIL_CN_ **TFSI** membrane showed an increase of at least two‐fold in water permeance, consistent with the self‐diffusion coefficient obtained from the MD simulation (Figure 4g). Whilst MXene/PIL_CN_ **OB** membrane maintained approximately the same permeance as that of the PIL_CN_ **Br**. This phenomenon could also be found with MXene/PIL membranes at a lower loading content (80 mg m^−2^) of MXene nanosheets (Figure S18). Given the similar *d*‐spacing for MXene/PIL_CN_ **TFSI** and MXene/PIL_CN_ **OB** (Figure 3b), their noticeable difference in water permeance could be ascribed to the hydrophilicity change of the MXene layer as discussed above. In addition, the rejection of both MXene/PIL_CN_ **TFSI** and MXene/PIL_CN_ **OB** increased to 99.0 %, demonstrating a satisfactory molecular sieving effect. It stemmed presumably from intercalation of larger‐size TFSI^−^ and OB^−^ into the MXene layer after the anion exchange, hindering the subsequent passage of dye molecules through 2D nanochannels. It was noteworthy that owing to the widest interlayer distance, the MXene/PIL_CN_ **TPB** membrane delivered the highest water permeance at the price of a lower rejection of 96.6 % (Figure 5b). Then, more comprehensive molecular transport properties in MXene/PIL_CN_ **TFSI** membrane were further investigated by filtering the aqueous solution containing organic dyes of different sizes and charges. As shown in Figure 5c, both high rejection and permeance for most dye molecules can be achieved by MXene/PIL_CN_ **TFSI**. As control experiment, the anion exchange treatment was also conducted to the pristine MXene membrane (without PILs) to highlight the unique function of PILs in the counter anion exchange process. Briefly, the same aqueous solution containing TFSI^−^, OB^−^, and TPB^−^ were flown through the pristine MXene membrane (referred as MXene‐TFSI, MXene‐OB, and MXene‐TPB), respectively. The water permeance increased slightly with the anion exchanged membranes that bear more hydrophobic TFSI^−^ or TPB^−^, while the MXene‐OB membrane with the less hydrophobic counter anion OB^−^ exhibited a compromised water permeance (Figure S19). Overall, it showed a similar but indistinctive trend as the PIL‐assembled MXene membrane, indicative of the central role of PILs in the effective counter anion exchange of MXene‐based membranes. These results manifested the effectiveness and benefits of anion exchange on the separation performance of PIL‐based MXene membranes with well‐chosen counter anions.

Overall, our MXene/PIL membranes exhibited a remarkable separation performance among the state‐of‐the‐art 2D material‐based membranes (Table S3). As an additional benefit, the MXene/PIL membranes delivered a promising performance stability for practical applications. Both MXene/PIL_CN_ **Br** and MXene/PIL_CN_ **TFSI** membranes showed no significant decrease in permeance in a 9‐cycle test (Figure S20), and a well‐maintained rejection to CR.

To explore the universality of the charge‐driven assembly with PILs and the related anion exchange reaction for MXene membranes, three more PILs of different chemical structures (chemical structures in Figure S21), i.e., with various distal functionalities in the alkyl substituent were applied to assembly MXene. It was found that severe aggregation and sedimentation occurred to the MXene nanosheets when assembling with PIL${}_{\mathrm{CONH}{}_{2}}$ **Br** and PIL_COOH_ **Br** (Figure S22), probably due to enhanced H‐bonding formation between the PIL chains and MXene nanosheets. By contrast, PIL_Ph_ **Br** with a phenyl ring as the terminal group shows a stable and homogeneous assembly with MXene nanosheets, as it has less chance to build up H‐bonds with MXene here. Likewise, it exhibited a significantly improved water permeance and stable rejection after anion exchange with TFSI^−^, similar to PIL_CN_ **Br** studied above (Figure 5d). This result stressed the importance of the cationic backbone structures of PILs for the MXene assembly, and the general applicability of the anion exchange concept.

In conclusion, we successfully engineered the hydrophilicity of nanochannels within MXene‐based membranes with PILs as tailor‐made surface modifier to assemble MXene laminates via electrostatic interactions. By varying counter anions in the PIL, the interlayer galleries were modulated from being hydrophilic to more hydrophobic. Both the permeance and rejection were significantly improved for MXene/PIL membrane after counter anion exchange from hydrophilic Br^−^ to more hydrophobic TFSI^−^. Equally important, the backbone of PILs also exhibited a crucial impact on the assembly of MXene nanosheets. This study paves the way for the future development of high‐performance MXene membranes via ion‐engineering of nanochannels for better molecular transport and sieving function.

## Conflict of interest

The authors declare no conflict of interest.

1

## Supporting information

As a service to our authors and readers, this journal provides supporting information supplied by the authors. Such materials are peer reviewed and may be re‐organized for online delivery, but are not copy‐edited or typeset. Technical support issues arising from supporting information (other than missing files) should be addressed to the authors.

Supporting Information

## Data Availability

Research data are not shared.
