# Supplementary material for: Poly(ionic liquid)‐Armored MXene Membrane: Interlayer Engineering for Facilitated Water Transport
Source: Angew Chem Int Ed Engl. 2022 May 3;61(27):e202202515. doi: 10.1002/anie.202202515 (PMC9324950; doi:10.1002/anie.202202515)
Supplement: Supplementary file 1 — Supporting Information [file ANIE-61-0-s001.pdf]

Angewandte Chemie
Eine Zeitschrift der Gesellschaft Deutscher Chemiker

Supporting Information

Poly(ionic liquid)-Armored MXene Membrane: Interlayer Engineering for Facilitated Water Transport

M. Yi, M. Wang, Y. Wang, Y. Wang*, J. Chang, A. K. Kheirabad, H. He, J. Yuan*, M. Zhang**

Poly(ionic liquid)-armored MXene Membrane: Interlayer Engineering for Facilitated Water Transport

Ming Yi[# a,b,c], Mi Wang[# d], Yan Wang *[a,b] Yanlei Wang [d] *, Jian Chang [c], Atefeh Khorsand Kheirabad [c], Hongyan He[d], Jiayin Yuan *[c], Miao Zhang *[c]

[a]Key Laboratory of Material Chemistry for Energy Conversion and Storage (Huazhong University of Science and Technology), Ministry of Education, Wuhan, 430074, P. R. China

[b]Hubei Key Laboratory of Material Chemistry and Service Failure, School of Chemistry and Chemical Engineering, Huazhong University of Science and Technology, Wuhan, 430074, P. R. China

[c]Department of Materials and Environmental Chemistry, Stockholm University, Stockholm, 10691, Sweden

[d]Beijing Key Laboratory of Ionic Liquids Clean Process, State Key Laboratory of Multiphase Complex Systems, Institute of Process Engineering, Chinese Academy of Sciences, Beijing 100190, P. R. China

Experimental section

Materials

All chemicals from chemical suppliers were used as received without any further purification. High purity (≥ 99%) Ti_3AlC_2 MAX phase powder (400 mesh) was ordered from Laizhou Kai Kai Ceramics Materials Co., Ltd. Hydrochloric acid (HCl, 37 %) was supplied from VWR International. Tetrahydrofuran (THF, ≥ 99.9%), dopamine hydrochloride, tris (hydroxymethyl) aminomethane (THAM, ≥ 99.8%), copper sulfate ($CuSO_4$, ≥ 99.99%), lithium fluoride (LiF, ≥ 99.98% trace metals basis), lithium bis(trifluoromethylsulfonyl)imide (LiTFSI, 99%), lithium bis(oxalate)borate (LiOB, ≥ 99%), sodium tetraphenylborate (NaTPB, 95%), Congo red (CR, ≥ 35%), triochrome black T (BT, indicator grade), methylene blue (MB, ≥ 97.0%), and Rose Bengal (RB, 95%) were purchased from Sigma-Aldrich. Nylon-66 commercial microfiltration membrane was purchased from Tianjing JinTeng Experiment Equipment Co., Ltd. Deionized (DI) water was used throughout the experiment unless particularly stated.

Synthesis of poly(ionic liquid)s

Poly(1-carboxamide-3-vinylimidazolium bromide) ($PIL_{CN}Br$)

1-Vinylimidazole (23.5 g, 0.25 mol) and bromoacetonitrile (30 g, 0.25 mol) were added sequentially into diethyl ether (55 mL), and the solution was stirred overnight at room temperature. The reaction mixture was then filtered off, washed three times with diethyl ether, and dried in a vacuum oven at 40 °C overnight. 1-Cyanomethyl-3-vinylimidazolium bromide was obtained as a white powder.

1-Cyanomethyl-3-vinylimidazolium bromide (10 g), and AIBN (200 mg) were added to DMSO (100 mL) placed in a 250 mL Schlenk flask. The reaction mixture was deoxygenated three times by a freeze-pump-thaw procedure. The reaction mixture was heated at 70 °C overnight. The reaction mixture was then precipitated into a 2 L THF/acetone mixture (v/v = 3:1), filtered off, washed with THF for three times, and then dried at 70 °C using a vacuum oven. Poly(1-cyanomethyl-3-vinylimidazolium bromide) was

obtained as a slightly yellow powder. The corresponding chemical structures were confirmed by ^{1}H-NMR spectrum in Figure S23.

Poly(1-carboxamide-3-vinylimidazolium bromide) ($PIL_{CONH2}Br$)

In a 100 mL flask, 1-vinylimidazolium (20.0 g, 0.21 mol), 2-bromoacetamide (34.86 g, 0.21 mol) and 2,6-di-tert-butyl-4-methylphenol (0.4 g, 1.815mmol) were added into 70 mL of acetone. After the mixture was stirred for 24 hours at 50 °C, the precipitate was filtered off and washed with diethyl ether and finally dried under vacuum at 40 °C overnight. 1-Carboxamide-3-vinylimidazolium bromide was obtained as a white powder. For the polymerization, 10 g of 1-carboxamide-3-vinylimidazolium bromide monomer, 200 mg of AIBN, and 100 mL of DMSO were loaded into a 250 mL flask. The mixture was deoxygenated three times by a freeze-pump-thaw procedure and finally charged with nitrogen. The reaction mixture was then placed in an oil bath at 75 °C for 24 h. When cooling down to room temperature, the reaction mixture was dropwise added to excess THF. The precipitate was filtered off, washed with excess of THF and dried at 60 °C under vacuum. Poly(1-carboxamide-3-vinylimidazolium bromide) was obtained as a white powder. The product was then characterized *via* ^{1}H-NMR spectroscopy (Figure S24).

Poly(1-carboxymethyl-3-vinylimidazolium bromide) ($PIL_{COOH}Br$)

In a 100 mL flask, 1-vinylimidazole (20.0 g, 0.21 mol) and bromoacetic acid (29.2 g, 0.21 mol) were added into 70.0 mL of acetone. After the mixture was stirred for 24 hours at room temperature, the precipitate was filtered off and washed with diethyl ether and finally dried under vacuum at 40 °C overnight. 1-Carboxymethyl-3-vinylimidazolium bromide was obtained as a white powder. For the polymerization, 10 g of 1-carboxymethyl-3-vinylimidazolium bromide monomer, 200 mg of AIBN, and 100 mL of DMSO were loaded into a 250 mL flask. The mixture was deoxygenated three times by a freeze-pump-thaw procedure and finally charged with nitrogen. The reaction mixture was then placed in an oil bath at 75 °C for 24 h. When cooling down to room temperature, the reaction mixture was dropwise

added to excess THF. The precipitate was filtered off, washed with excess of THF and dried at 80 °C under vacuum. Poly(1-carboxymethyl-3-vinylimidazolium bromide) was obtained as a white powder. The product was then characterized *via* ^{1}H-NMR spectroscopy (Figure S25).

Poly(1-benzyl-3-vinylimidazolium bromide) ($PIL_{Ph}Br$)

1-Vinylilimidazole (18.82 g, 0.20 mol) and benzyl bromide (34.2 g, 0.22 mol) were dissolved into 40 mL methanol. The reaction was conducted at room temperature for three hours and then temperature increased to 50 °C for overnight. After that, the solution was added to diethyl ether dropwise. The reaction mixture was then filtered off, washed three times with diethyl ether, and dried in a vacuum oven at 40 °C overnight. 1-Benzyl-3-vinylimidazolium bromide was obtained as a white powder. For the polymerization, 10 g of 1-benzyl-3-vinylimidazolium bromide and 200 mg of AIBN were dissolved into 100 mL of DMF and were placed into a 250 mL Schlenk flask. The reaction mixture was deoxygenated three times by a freeze-pump-thaw procedure. The reaction mixture was heated at 70 °C overnight. Poly(1-benzyl-3-vinylimidazolium bromide) as a yellowish power was obtained *via* precipitating the reaction mixture into THF. The product was then characterized *via* ^{1}H-NMR spectroscopy (Figure S26).

Synthesis of MXene

MXene dispersion was synthesized following the minimally intensive layer delamination (MILD) method as reported previously,[1] with minor modification. In detail, 1.5 g of high-purity MAX power (equivalent of 12 M) was cautiously added into a mixture of 30 mL of 9 M HCl and 2.4 g of LiF under continuous stirring over the course of 10 min in an ice bath. The etching reaction was stirred and kept for 36 h at room temperature. Then the multilayer Ti_3AlC_2 and unetched MAX particles were washed and centrifuged (at 10000 rpm one hour for each cycle) with DI water until the supernatant reached a pH value of 6. $Ti_3C_2T_x$ MXene sheet suspension was produced by the exfoliation process of resultant slurry under

sonication for 1 h under a N_2 flow environment. The concentration of MXene suspension was obtained by calculating the weight of dry MXene sheets.

PDA coating of nylon-66 substrate

Dopamine hydrochloride (2 mg/mL) was dissolved in an aqueous solution (pH = 8.5, 5 mM) containing tris(hydroxymethyl) aminomethane (THAM) and $CuSO_4$ (5 mM). A nylon-66 substrate was immersed in the dopamine THAM buffer solution for 3 h at 40 °C. Subsequently, it was washed by DI water to remove the residual PDA solution and then stored in DI water prior to use.

Fabrication of MXene/PILs membranes

First, 140 mg of PIL_{CN}**Br** power was dissolved in 60 mL DI water solution, 40 mL 0.875 mg/mL MXene suspension was then added dropwise into the PIL_{CN}**Br** solution for a 12-h mixing at room temperature to allow the assembly of MXene nanosheets. Next, the MXene/PIL_{CN}**Br** suspension was obtained after removing the residential PIL_{CN}**Br** by three-times centrifugation (10000 rpm). Afterwards, the pristine and PIL-assembled MXene membranes with different loadings were prepared by filtering the corresponding suspensions through a polydopamine-coated porous nylon-6 substrate. In this process, a vacuum filtration (0.1 MPa) treatment was applied to assist the filtering of various MXene suspensions, and the diameter of the used Buchner funnel for filtration was 40 mm. The resultant membranes were denoted as MXene and MXene/PIL_{CN}**Br**. In the anion exchange step, 100 mL of aqueous solutions of LiTFSI, LiOB, or NaTPB (chemical structure in Figure S4) at a concentration of 1.7 mM were vacuum-filtrated throughout the MXene/PIL_{CN}**Br** membrane to allow the anion exchange of MXene/PIL_{CN}**Br** membrane. The corresponding membranes were referred as MXene/PIL_{CN}**TFSI**, MXene/PIL_{CN}**OB**, and MXene/PIL_{CN}**TPB**, respectively. As control experiments, LiTFSI solution was directly added into a mixed solution of MXene and PIL_{CN}**Br**, then the mixture dispersion was sonicated and deposited onto a substrate of the same type *via* filtration, which formed a membrane, termed MXene@PIL_{CN}**TFSI**. Note

that we kept the mass of MXene constant in different membranes instead of the total mass of the MXene/PIL composite, which means a higher loading of MXene contains more PILs in MXene laminates. In addition, another set of control experiment was also carried out. Briefly, anion exchange was conducted to pristine MXene membrane. Briefly, aqueous solution containing $TFSI^-$, OB^-, and TPB^- (1.7 mM) were flown through the pristine MXene membrane, respectively. The corresponding membranes were (referred as MXene-TFSI, MXene-OB, and MXene-TPB, respectively.

Characterizations

^{1}H-NMR spectra were recorded at room temperature using a Bruker DPX-400 spectrometer operating at 400 MHz. D_2O and DMSO-d_6 was used as a solvent for the measurement. The morphology of the membrane was characterized by scanning electron microscopy (SEM, JEOL 7000, Japan) operated at 5 kV, and the element distribution was detected with the energy dispersive X-ray spectroscopy (EDX) of SEM. The transmission electron microscopy (TEM, JEOL JEM-2100F, Japan) and selected area electron diffraction (SAED) studies were performed under an accelerating voltage of 100 kV. Atomic force microscopy (AFM, Veeco Instruments, CA) was conducted utilizing a Nanoscope V in tapping mode. MXene/PIL_{CN}**Br** suspension after washing was freeze-dried into power, the thermo gravimetric analysis (TGA, TA Instruments Discovery TG) was then applied to detect the grafting degree of PILs to the MXene nanosheets. X-ray diffraction (XRD, Bruker, D8 Advance, America) patterns of the membrane were collected using Cu-K_α radiation at 40 kV and 40 mA in the 2θ angle range of 5° to 90°, with the scan step of 0.02°. Zeta potential and dynamic light scattering (DLS) measurements of MXene/PIL_{CN}**Br** suspension after washing were performed on a Zetasizer (Malvern, England). X-ray photoelectron spectroscopy (XPS, Thermo Fisher, Escalab 250XI, America) analysis was carried out using a monochromatic Al-K_α X-ray source (hυ = 1486.6 eV) operated at 150 W. Inductively coupled plasma optical emission spectrometry (ICP-OES, iCAP7200plus, China) was applied for the determination of S content. The chemical digestion of a free-standing MXene/PIL_{CN}**TFSI** membrane was completed in the

mixture of 40 wt% HNO_3 and 30 wt% H_2O_2 (v/v = 10/1) at 80 ºC for 1 h. Contact angle to both water and methylene iodide was measured on a Geniometer (Krüss, DSA25, Germany) at ambient conditions. The surface energy of membranes studied here was evaluated by the Owens−Wendt method,[2] based on the experimentally determined intrinsic contact angles of water (θ_{water} with γ_{water} = 72.8 mN/m) and methylene iodide (θ_{MI} with γ_{MI} = 50.8 mN/m),

$$\sqrt{\gamma_{water}^{d}}\sqrt{\gamma_{sv}^{d}} + \sqrt{\gamma_{water}^{p}}\sqrt{\gamma_{sv}^{p}} = \frac{(1+\cos(\theta_{water}))\gamma_{water}}{2} \quad (1)$$

$$\sqrt{\gamma_{MI}^{p}}\sqrt{\gamma_{sv}^{p}} = \frac{(1+\cos(\theta_{MI}))\gamma_{MI}}{2} \quad (2)$$

where the dispersive (γ_{water}^{d} = 21.8 mN/m) and polar components (γ_{water}^{p} = 21.8 mN/m) of water, and those of methylene iodide (γ_{MI}^{d} = 49.5 mN/m and γ_{MI}^{p} = 1.3 mN/m), were taken from literature.[3]

Details of molecular dynamics simulation

$Ti_3C_2T_x$ MXene nanosheet models comprised of PIL_{CN}**Br** or PIL_{CN}**TFSI** and H_2O molecules stuck in MXene slabs were displayed in Figure 1a. The size of the MXene nanosheet is 6.4 × 3.1 nm^2, and the detailed number of PIL and water molecules are summarized in Table S4. Periodic boundary condition (PBC) was applied along with the MXene slab, while an open boundary condition was employed through the slab. Nonpolarizable all-atom optimized potentials for the liquid simulation (OPLS-AA) force field[4] was applied to describe IL interactions, which have been extensively utilized in studying the structure and property of ionic liquids. The TIP3P model[5] was implemented for water. Atomic charges and force field parameters for $Ti_3C_2T_x$ MXene are listed in Table S5. The short-range electrostatic interactions are calculated using Coulomb pair interactions, while long-range terms use particle-particle particle-mesh (PPPM) solver. The *vdW* term is computed using 12-6 Lennard-Jones potential, and the geometric mixing rule was used to model the parameters. The cut-off distance was set to 1.2 nm for both electrostatic and vdW terms.

All modeling and simulations were operated at 300 K using the large-scale atomic/molecular massively parallel simulator (LAMMPS).[6] The timestep is 2 fs. After a 10 ns simulation, the MXene/PILs system

will reach the equilibrated state, 5-ns-long simulations are conducted continuously, where the mass density distribution and coordination analysis will be performed. The molecular diffusion coefficient D was calculated from the molecular trajectories of water using Einstein's definition, where at least 5 independent simulation runs were implemented to obtain the averaging results.

NF performance tests of MXene-based membranes

The separation performance of the membrane was evaluated using a lab-scale cross-flow NF filtration setup (Suzhou Faith & Hope Membrane Technology Co. Ltd.) with an effective area of 10.75 cm^2. Before testing, the membrane was pre-filtrated with DI water under the operational pressure of 2 bar, a crossflow rate of 40 L/h, until the flux reached a steady state. The permeance was then collected at 1 bar. Various dye solutions (CR, BT, RB, and MB) at a concentration of 100 ppm were employed as the feed solution for the tests. The permeability of the membrane was calculated using the following equation:

$$\text{Permeability} = \frac{V}{A \times t \times P} \quad (1)$$

where *V* is the permeate volume (L); *A* is the effective membrane area (m^2); *t* is the filtration time (h). *P* is the operating pressure, fixed at 1 bar.

The dye rejection (*R*) was calculated as the following equation:

$$\text{Rejection} = \left(1 - \frac{C_p}{C_f}\right) \times 100 \quad (2)$$

where C_f and C_p are the dye concentrations in the feed and permeance, respectively. A UV-Vis spectrophotometer (Thermo Fisher, GENESYS 150, America) was employed to measure the dye concentration.

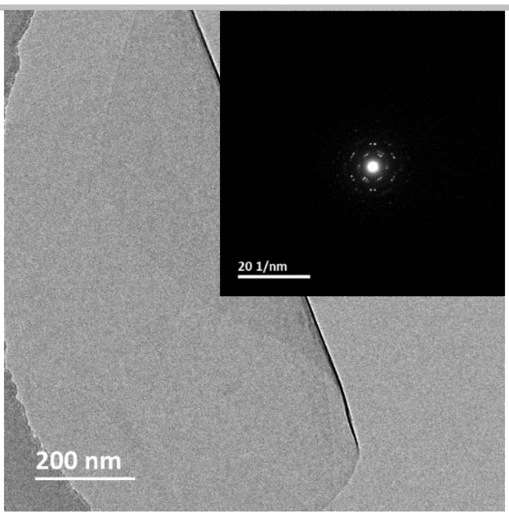

Figure S1. HRTEM image (inset shows the SAED pattern) of pristine MXene nanosheets.

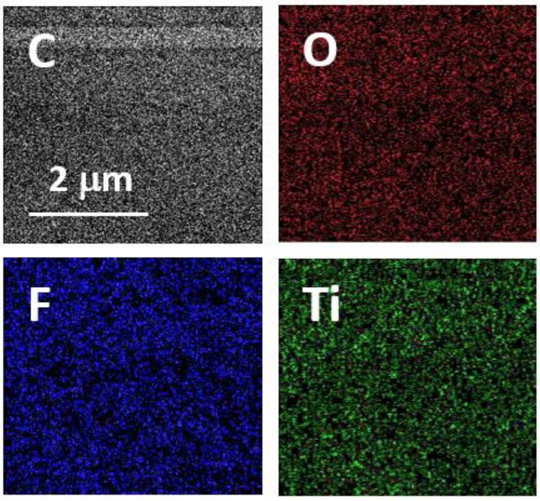

Figure S2. EDS mapping of pristine MXene membrane.

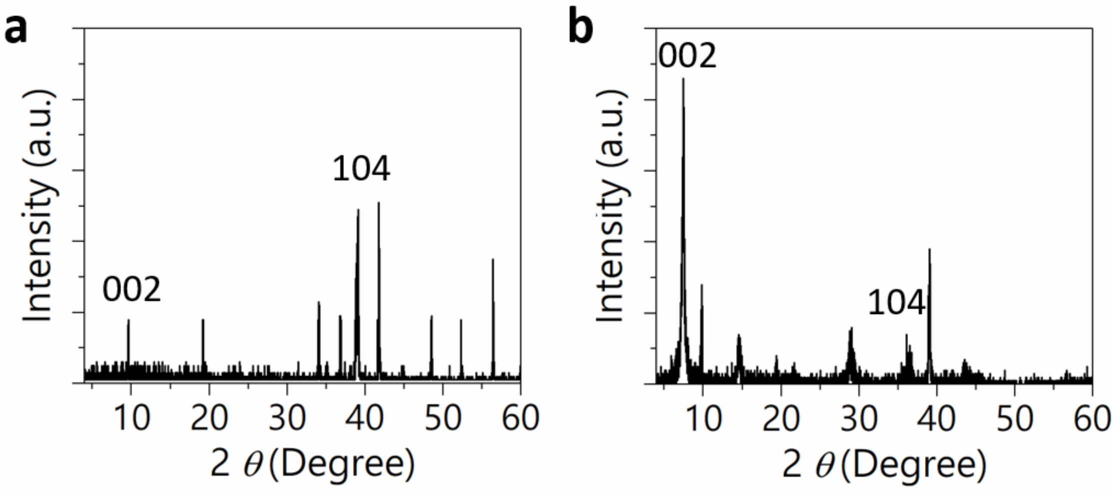

Figure S3. XRD patterns of (a) bulk Ti_3AlC_2 (MAX) and (b) $Ti_3C_2T_x$ MXene nanosheets.

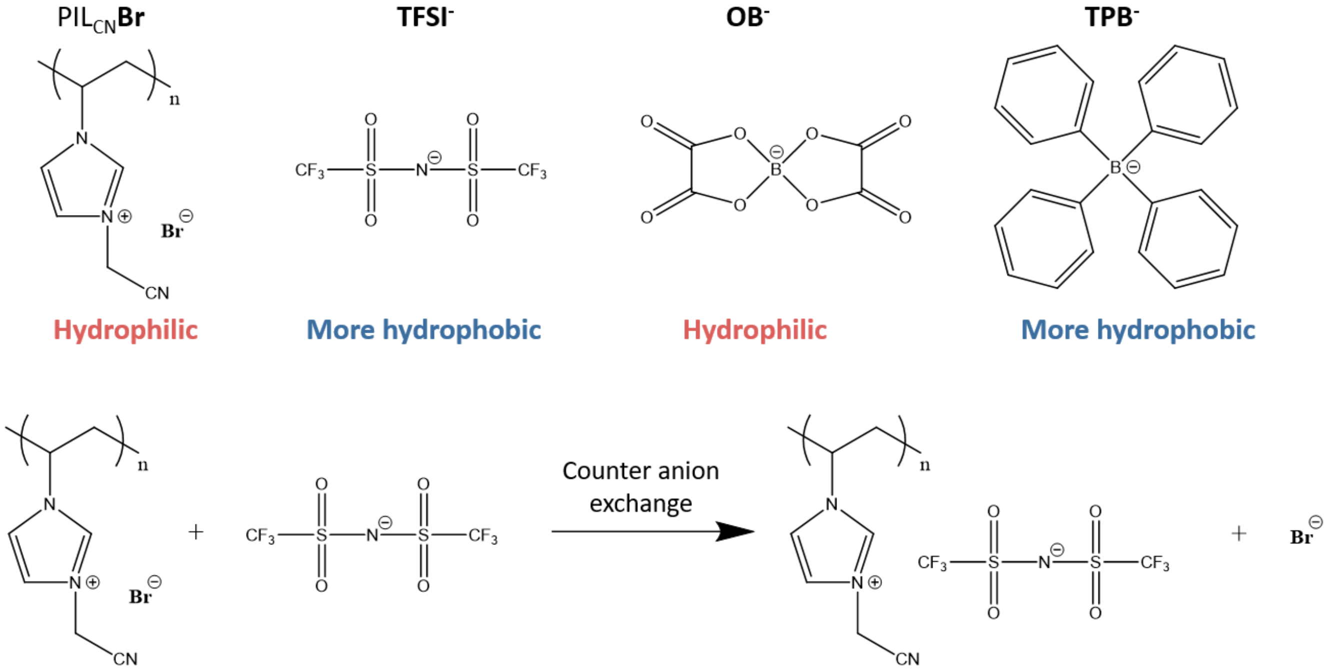

Figure S4. Chemical structures and anion exchange mechanism of PILs and various counter anions.

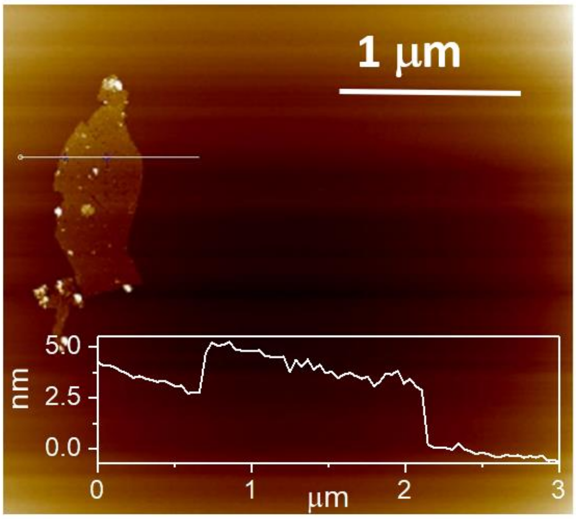

Figure S5. AFM image of MXene/PIL$_{CN}$**Br** MXene nanosheets. Inset is the height profile.

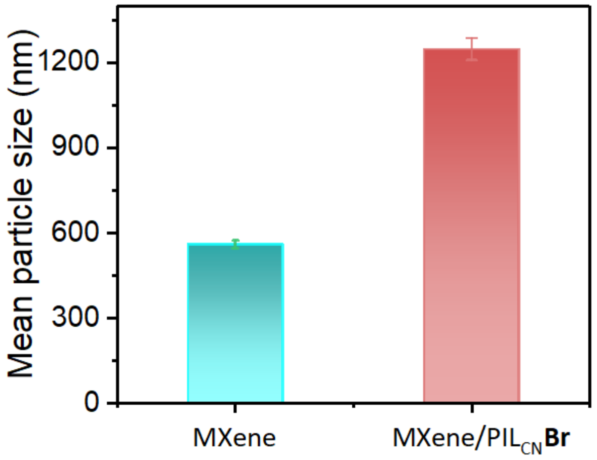

Figure S6. Mean particle sizes of the pristine MXene and the MXene/PIL$_{CN}$**Br** nanosheets.

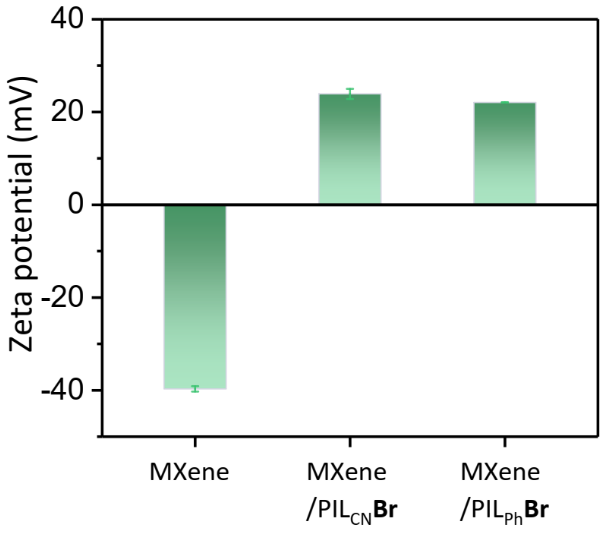

Figure S7. Zeta potentials of pristine MXene and PIL-assembled MXene nanosheets suspensions.

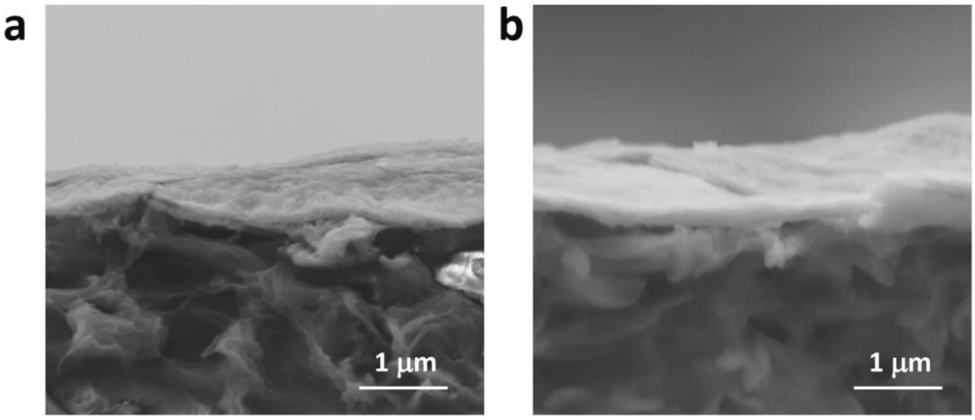

Figure S8. Cross-sectional SEM images of (a) pristine MXene and (b) MXene/PIL_{CN}**Br** membranes (MXene loading: 160 mg m^{-2}).

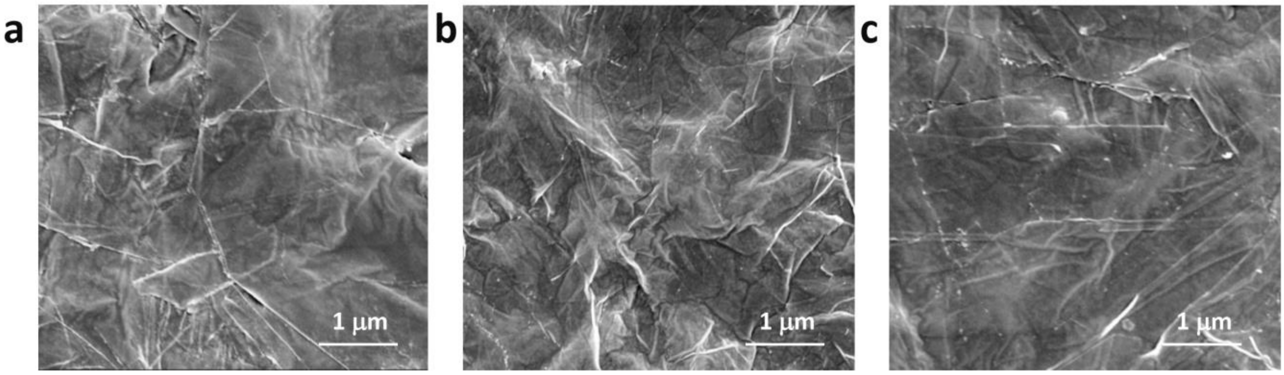

Figure S9. Surface SEM images of (a) pristine MXene, (b) MXene/PIL_{CN}**OB**, and (C) MXene/PIL_{CN}**TPB** membranes.

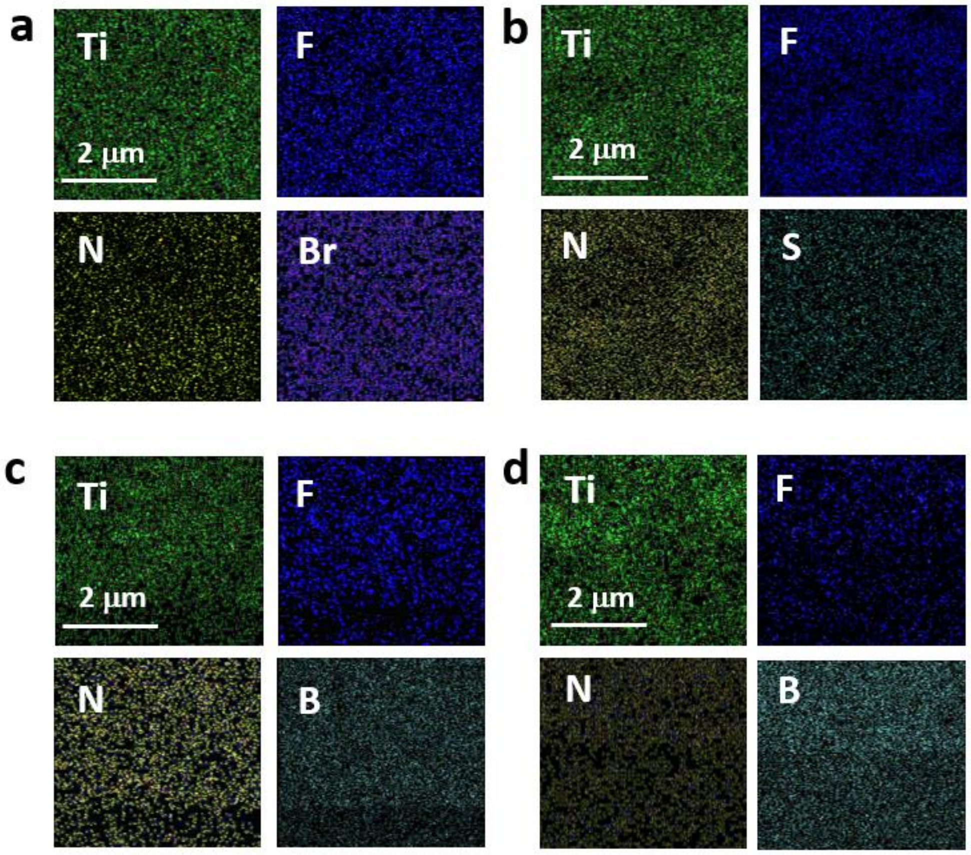

Figure S10. EDS mapping of (a) MXene/PIL_{CN}**Br**, (b) MXene/PIL_{CN}**TFSI**, (c) MXene/PIL_{CN}**OB**, and (d) MXene/PIL_{CN}**TPB** membranes.

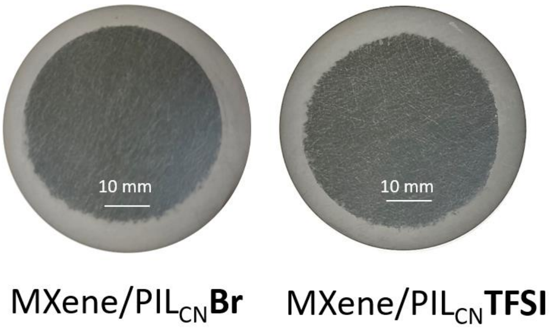

Figure S11. Digital photographs of the MXene/PIL_{CN}**Br** and MXene/PIL_{CN}**TFSI** membranes.

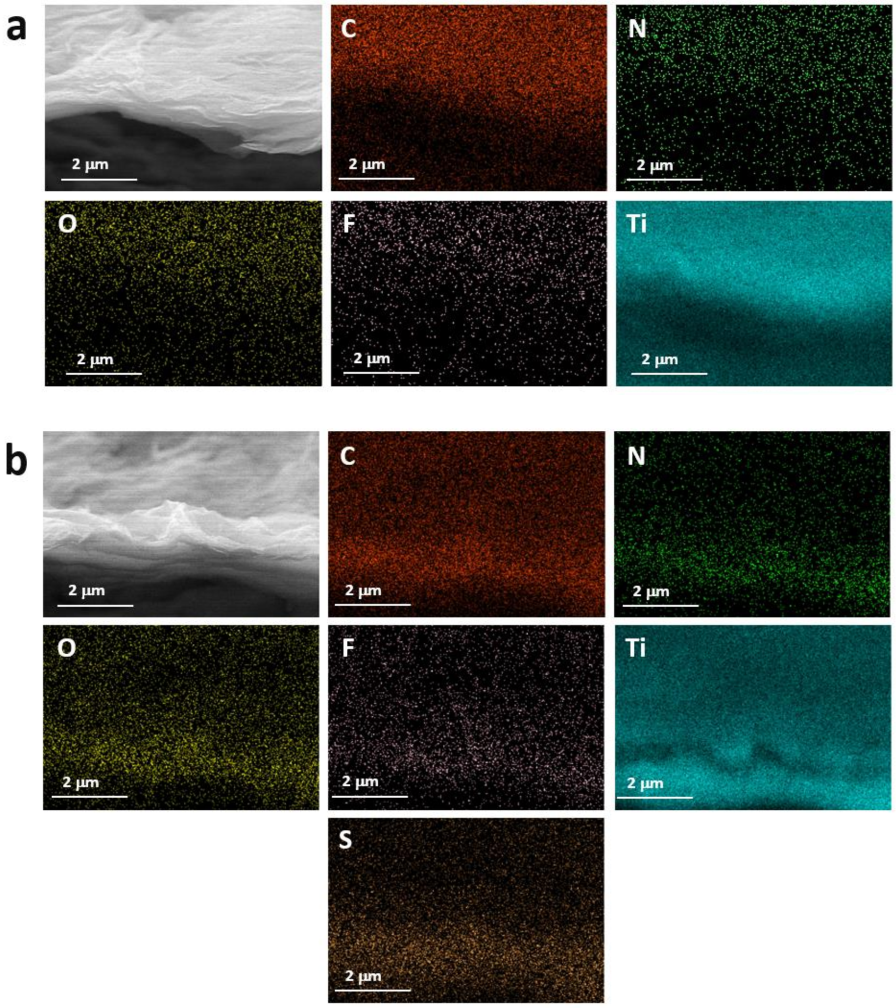

Figure S12. Cross-sectional EDS mapping of the MXene/PIL$_{CN}$**Br** and MXene/PIL$_{CN}$**TFSI** membranes. (MXene/PIL$_{CN}$**Br** and MXene/PIL$_{CN}$**TFSI** membranes with significantly thicker layer were prepared here for a better observation of cross-sectional mapping)

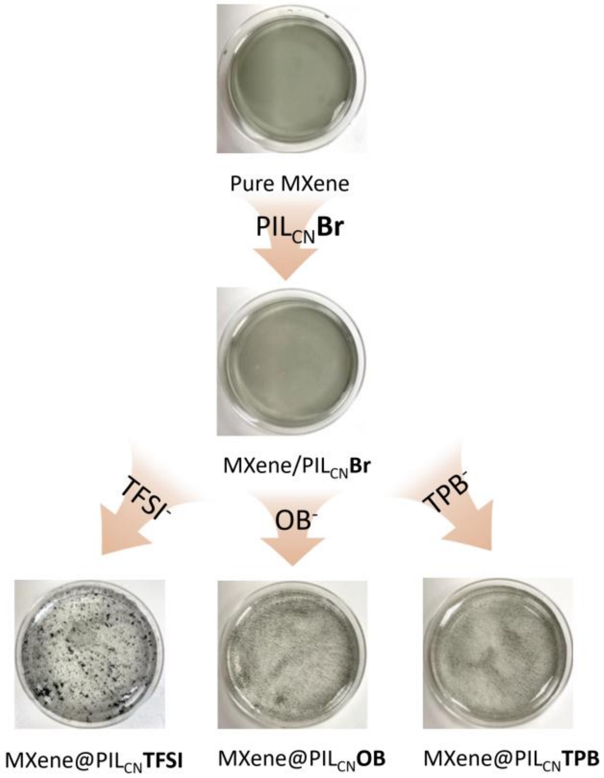

Figure S13. The phenomenon of directly adding counter anions solution into MXene/PIL$_{CN}$**Br** suspension.

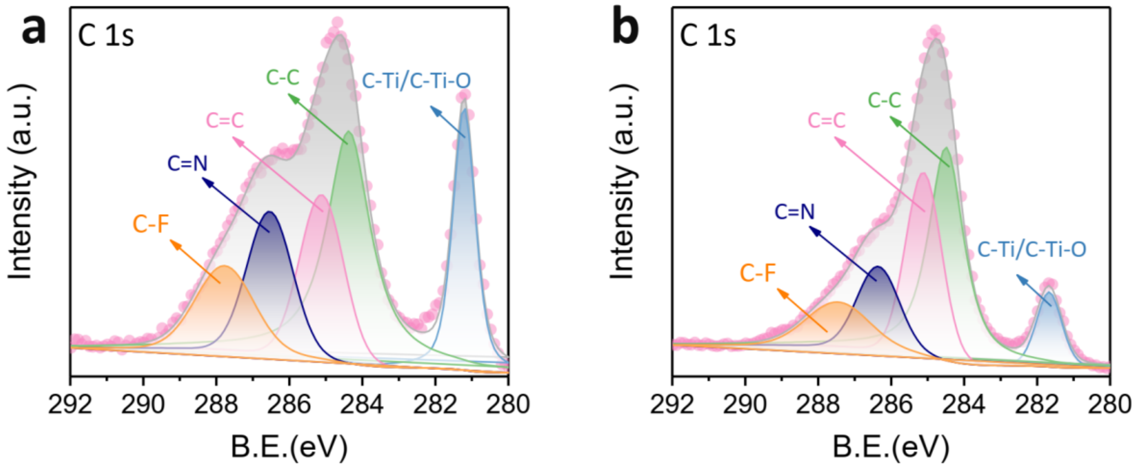

Figure S14. Peak deconvolution of narrow-scan spectra of C 1s for (a) MXene/PIL$_{CN}$**OB** and (b) MXene/PIL$_{CN}$**TPB** membrane.

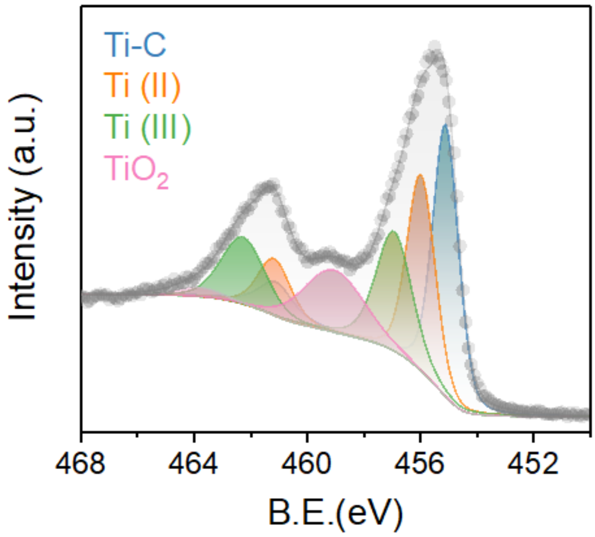

Figure S15. Peak deconvolution of narrow-scan spectrum of Ti 2p for MXene/PIL$_{CN}$**TFSI** membrane.

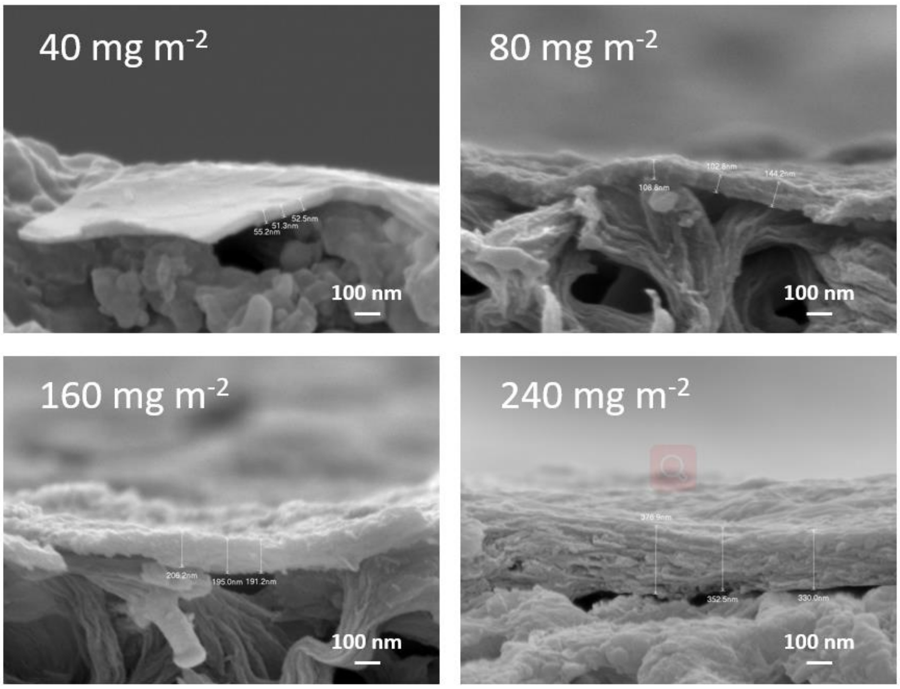

Figure S16. The cross-sectional images of membranes with different loading amount of MXene/PIL$_{CN}$**Br** nanosheets.

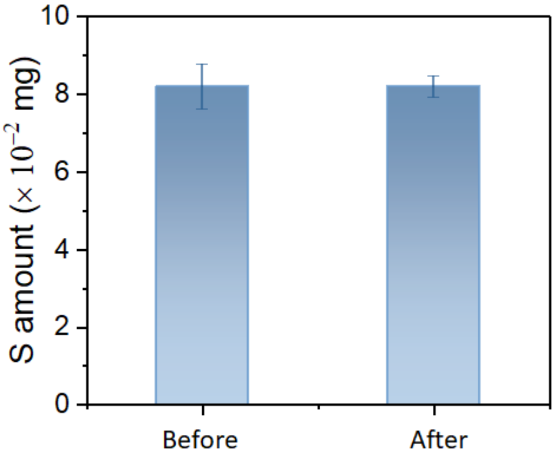

Figure S17. The amount of S element detected by ICP-OES in MXene/PIL membranes before and after NaCl filtration. (Digested membrane weight: 13 mg)

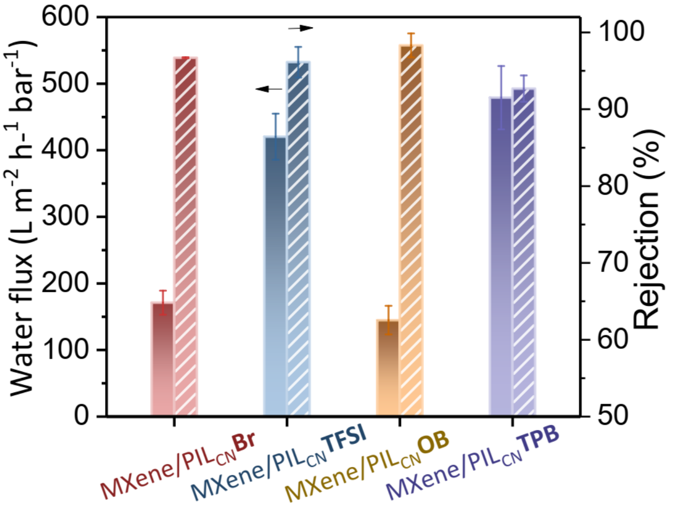

Figure S18. Separation performance of MXene, MXene/PIL_{CN}**Br** and various anion exchanged MXene membranes to CR (MXene loading: 80 mg m^{-2})

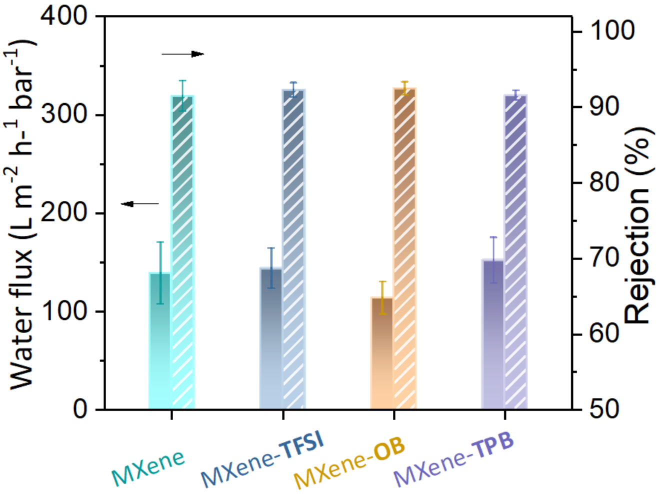

Figure S19. Separation performance of the pristine MXene membranes bearing various anions towards CR. (MXene loading: 160 mg m^{-2})

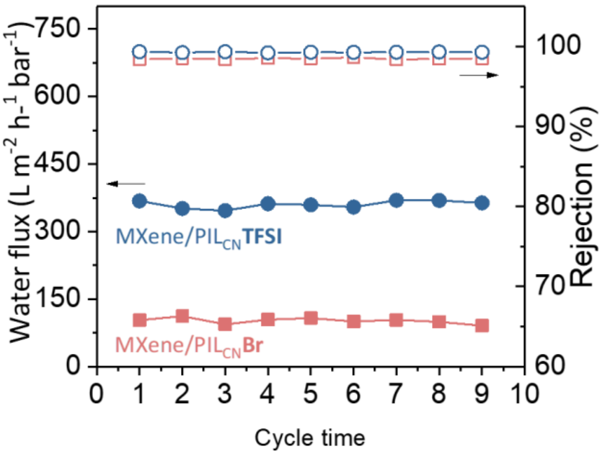

Figure S20. The stability test of separation performance *vs.* cycle times for MXene/PIL membranes. (Each cycle for 15-min filtration)

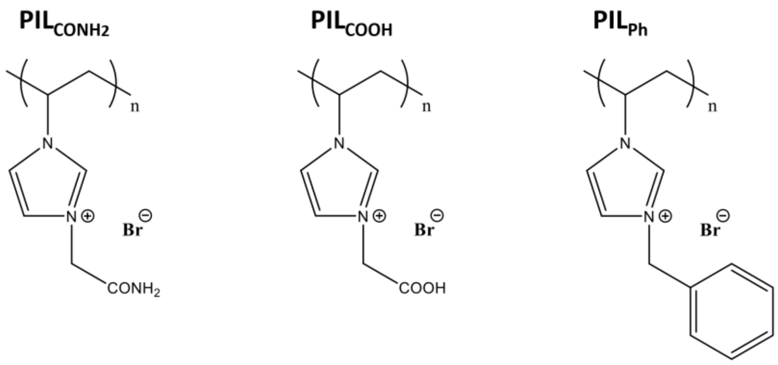

Figure S21. The chemical structures of PILs with different terminal groups in the alkyl substituent.

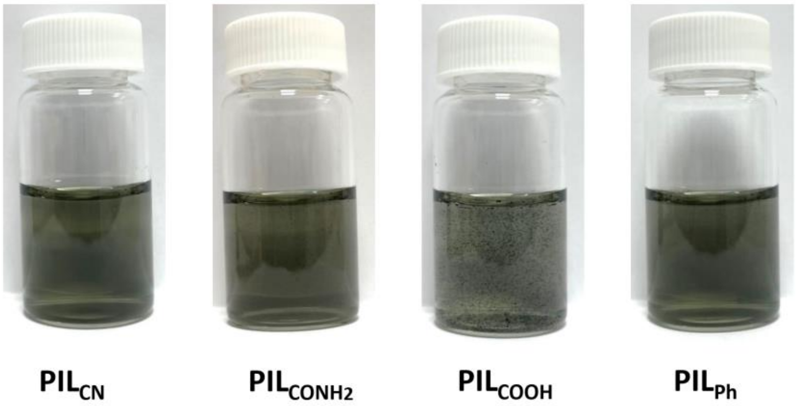

Figure S22. The MXene suspensions assembled with various types of PILs.

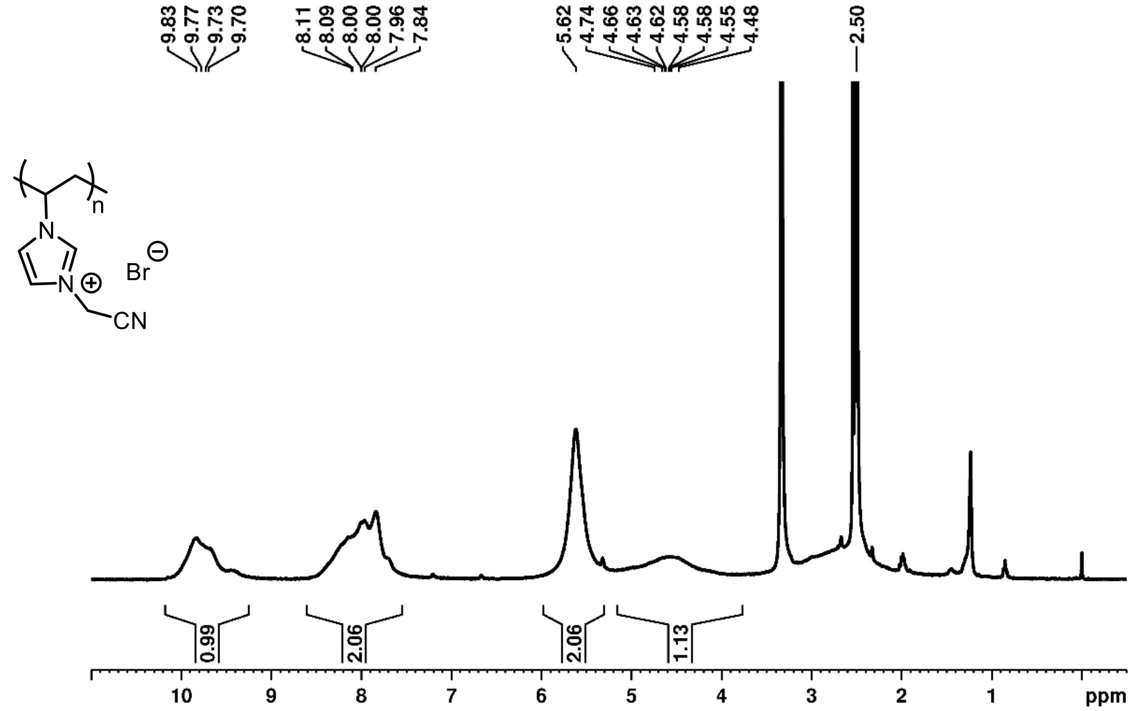

Figure S23. ^{1}H-NMR spectrum of PIL_{CN}**Br** in DMSO-d_6.

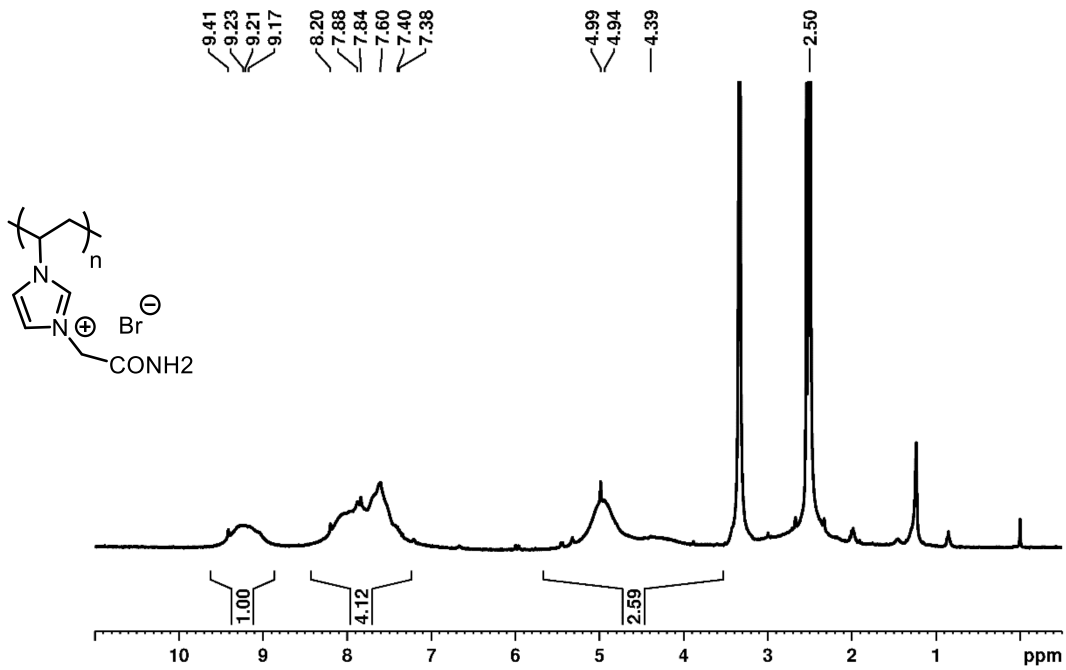

Figure S24. ^{1}H-NMR spectrum of PIL$_{CONH_2}$**Br** in DMSO-*d*$_6$.

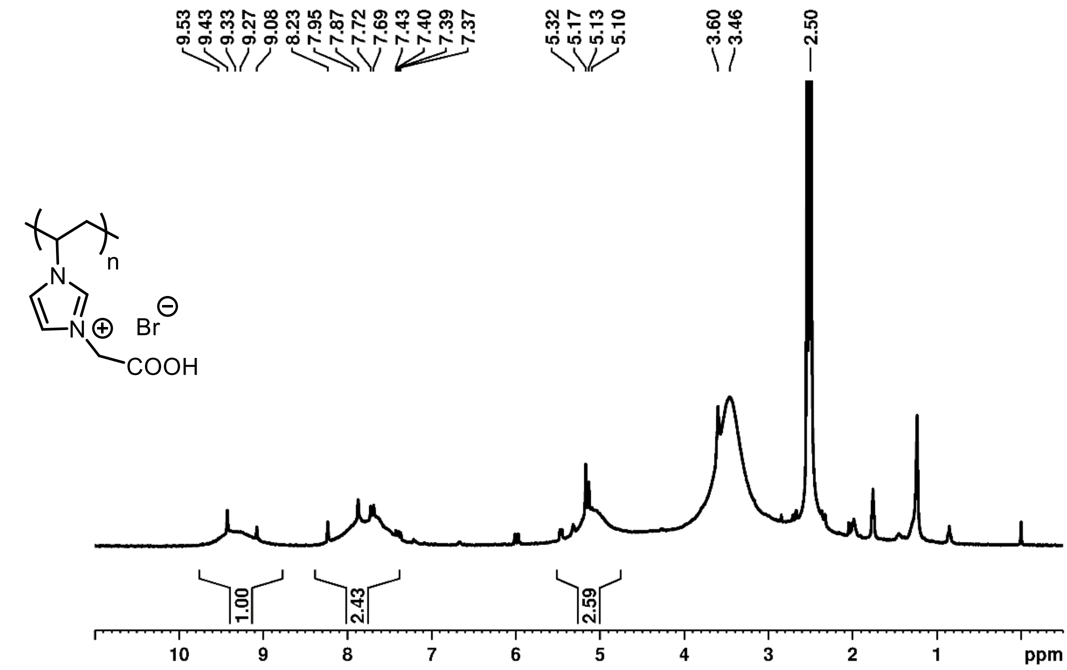

Figure S25. ^{1}H-NMR spectrum of PIL$_{COOH}$**Br** in DMSO-*d*$_6$.

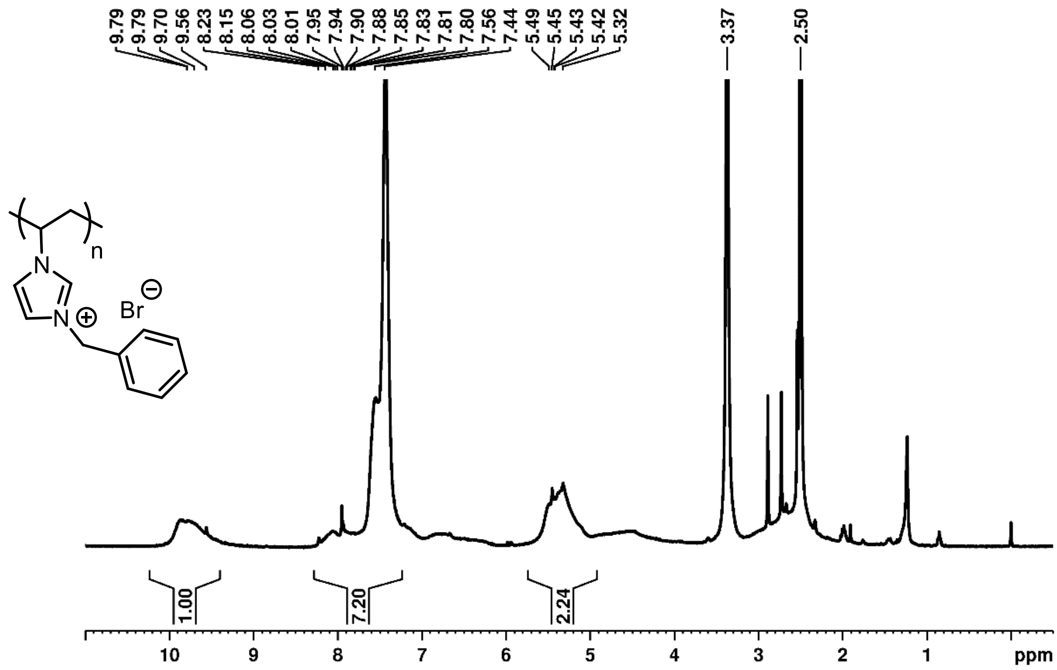

Figure S26. ^{1}H-NMR spectrum of PIL_{Ph}**Br** in DMSO-d_6.

Table S1. Surface elemental composition of the MXene, MXene/PIL_{CN}**Br** and various anion exchanged MXene membranes by XPS analysis.

Membrane	Atomic percentage (%)							
	Ti 2p	O 1s	C 1s	F 1s	N 1s	Br 3d	S 2p	B 1s
MXene	26.8	18.2	44.19	10.8	/	/	/	/
MXene/PILs-Br	20.8	16.53	47.04	5.89	8.81	0.92	/	/
MXene/PILs-TFSI	17.76	16.4	46.09	8.96	9.6	/	1.18	/
MXene/PILs-BOB	16.34	18.14	49.55	6.38	8.64	/	/	0.95
MXene/PILs-BBenz	16.31	17.23	52.4	4.84	8.46	/	/	0.76

Table S2. Surface elemental composition of MXene, MXene/PIL_{CN}**Br** and various anion exchanged MXene membranes by EDS analysis.

Membrane	Atomic percentage (%)							
	Ti K	O K	C K	F K	N K	Br L	S K	B K
MXene	1.26	16.76	80.95	1.03	/	/	/	/
MXene/PIL_{CN}**Br**	1.15	11.94	78.07	1.41	6.4	1.03	/	/
MXene/PIL_{CN}**TFSI**	1.39	11.84	78.01	1.01	6.44	/	1.32	/
MXene/PIL_{CN}**OB**	0.84	8.01	77.38	1.32	4.47	/	/	7.98
MXene/PIL_{CN}**TPB**	0.72	6.98	80.73	0.55	2.55	/	/	8.47

Note: The signal of Ti element was overlapped with that of O, thus low atomic percentage of Ti.

Table S3. Benchmarking of 2D materials-based membranes for dyes nanofiltration.

Membrane type	Permeance (L/m^2 h bar)	Dye rejection(%)	Molecular weight (g/mol)	Feed conc. (mg/L)	Ref.
g-C_3N_4	27.5	75.5	1111.1	/	[7]
Nanostrand Channeled WS_2 Membrane	704	82	960.8	/	[8]
MoS_2	245	89	960.8	125	[9]
TPTGCl@CNFS (COF)	100	78	319.9	100	[10]
NLC-GO	279	87	1111.1	10	[11]
GO	71	95	319.9	10	[12]
rGO	21.8	99.9	1111.1	13.5	[13]
Mxene	1084	90	960.8	/	[14]
GO/Mxene	16.7	98.6	319.9	10	[15]
Mxene-230	2302	96.3	1111.1	/	[16]
rGO/PDA/MXene	174.2	95	319.9	20	[17]
CNT/Mxene	340.5	95.1	514.4	100	[18]
PA/Mxene	508.6	98.3	416.4	100	[19]
MXene/PIL_{CN}**TFSI**	420.4	96.1	696.7	100	This work
	350.1	99.5	461.5	100	This work
MXene/PIL_{CN}**TPB**	478.9	92.7	696.7	100	This work

Table S4. The number of PILBr/TFSI/OB and water molecules in the simulation box.

	PIL	anion	H_2O
Br^-	8	80	150
$TFSI^-$	5	50	100
OB	5	50	75

Table S5. The 12-6 L-J potential parameters and charge of atoms for $Ti_3C_2T_x$ MXene in the MD simulations.

Atom	ε (kcal/mol)	σ (Å)	q (e)
Ti1	0.0348	1.9565	1.38
Ti2	0.0348	1.9565	1.54
C	0.0038	3.5001	-1.60
O	0.1700	3.0000	-1.21
H	0.0130	1.0980	0.58

References

[1] H. Chen, Y. Wen, Y. Qi, Q. Zhao, L. Qu, C. Li, *Adv. Funct. Mater.* **2019**, *30*, 1906996.

[2] D. K. Owens, R. C. Wendt, *J. Appl. Polym. Sci.* **1969,** *13*, 1741-1747.

[3] C. Boo, J. Lee, M. Elimelech, *Environ. Sci. Technol.* **2016**, *50*, 8112-8119.

[4] a) Y. Wang, C. Qian, F. Huo, J. Qin, H. He, *J. Mater. Chem. A* **2020**, *8*, 19908-19916; b) Z. Gan, Y. Wang, M. Wang, E. Gao, F. Huo, W. Ding, H. He, S. Zhang, *J. Mater. Chem. A* **2021**, *9*, 15985-15992; c) B. Li, C. Wang, Y. Zhang, Y. Wang, *Green Energy Environ.* **2021**, *6*, 253-260; d) M. Wang, Y. Wang, C. Wang, Z. Gan, F. Huo, H. He, S. Zhang, *J. Phys. Chem. Lett.* **2021**, *12*, 6078-6084.

[5] S. Gao, Z. Li, Y. Yang, Z. Wang, Y. Wang, S. Luo, K. Yao, J. Qiu, H. Wang, L. Cao, Z. Lai, J. Wang, *Acs Appl. Mater. Interfaces* **2021**, *13*, 36507-36516.

[6] a) S. Plimpton, *J. Comput. Phys.* **1995**, *117*, 1-19; b) Y. Wang, Z. Qin, M. J. Buehler, Z. Xu, *Nat. Commun.* **2016**, *7*, 12854; c) C. Wang, Y. Wang, Z. Gan, Y. Lu, C. Qian, F. Huo, H. He, S. Zhang, *Chem. Sci.* **2021**, *12*, 15503-15510.

[7] Y. Wang, L. Li, Y. Wei, J. Xue, H. Chen, L. Ding, J. Caro, H. Wang, *Angew. Chem. Int. Ed. Engl.* **2017**, *56*, 8974-8980.

[8] L. W. Sun, Y. L. Ying, H. B. Huang, Z. G. Song, Y. Y. Mao, Z. P. Xu, X. S. Peng, *ACS Nano*. **2014**, *8*, 6304-6311.

[9] L. Sun, H. Huang, X. Peng, *Chem. Commun.* **2013**, *49*, 10718-10720.

[10] H. Yang, L. Yang, H. Wang, Z. Xu, Y. Zhao, Y. Luo, N. Nasir, Y. Song, H. Wu, F. Pan, Z. Jiang, *Nat. Commun.* **2019**, *10*, 2101.

[11] A. Akbari, P. Sheath, S. T. Martin, D. B. Shinde, M. Shaibani, P. C. Banerjee, R. Tkacz, D. Bhattacharyya, M. Majumder, *Nat. Commun.* **2016**, *7*, 10891.

[12] H. Huang, Z. Song, N. Wei, L. Shi, Y. Mao, Y. Ying, L. Sun, Z. Xu, X. Peng, *Nat. Commun.* **2013**, *4*, 2979.

[13] K. Goh, W. C. Jiang, H. E. Karahan, S. L. Zhai, L. Wei, D. S. Yu, A. G. Fane, R. Wang, Y. Chen, *Adv. Funct. Mater*. **2015**, *25*, 7348-7359.

[14] L. Ding, Y. Y. Wei, Y. J. Wang, H. B. Chen, J. Caro, H. H. Wang, *Angew. Chem. Int. Ed. Engl.* **2017**, *56*, 1825-1829.

[15] S. C. Wei, Y. Xie, Y. D. Xing, L. C. Wang, H. Q. Ye, X. Xiong, S. Wang, K. Han, *J. Membr. Sci.* **2019**,

582, 414-422.

[16] J. Wang, P. Chen, B. Shi, W. Guo, M. Jaroniec, S. Z. Qiao, *Angew. Chem. Int. Ed. Engl.* **2018**, *57*, 6814-6818.

[17] X. F. Feng, Z. X. Yu, R. X. Long, Y. X. Sun, M. Wang, X. H. Li, G. Y. Zeng, *Sep. Purif. Technol.* **2020**, *247*.

[18] M. Ding, H. Xu, W. Chen, Q. Kong, T. Lin, H. Tao, K. Zhang, Q. Liu, K. Zhang, Z. Xie, *J. Mater. Chem. A* **2020**, *8*, 22666-22673.

[19] M. Yi, F. Heraly, J. Chang, A. Khorsand Kheirabad, J. Yuan, Y. Wang, M. Zhang, *Chem. Commun.* **2021**, *57*, 6245-6248.
